# Synthesis and Structure–Activity Relationship Study of 2-(amino)quinazolin-4(3*H*)-one Derivatives as Potential Inhibitors of Methicillin-Resistant *Staphylococcus aureus* (MRSA)

**DOI:** 10.3390/antibiotics14100967

**Published:** 2025-09-25

**Authors:** Jun Young Lee, Hyunjung Lee, Sungmin Kim, Jihwan Gim, Yunmi Lee, Chae Jo Lim, Hyun-Seob Song, Hyeung-geun Park, Soojin Jang, Chul Min Park

**Affiliations:** 1 Infectious Diseases Therapeutic Research Center, Korea Research Institute of Chemical Technology, 141 Gajeong-ro, Yuseong-gu, Daejeon 34114, Republic of Korea; ljy3695@krict.re.kr (J.Y.L.); ksm0191@krict.re.kr (S.K.);; 2 Research Institute of Pharmaceutical Sciences, College of Pharmacy, Seoul National University, Seoul 08826, Republic of Korea; 3 Antibacterial Resistance Laboratory, Institut Pasteur Korea, Seongam-si 13488, Republic of Korea; hyunjung.lee@ip-korea.org (H.L.); yunmi.lee@ip-korea.org (Y.L.); 4 Data Convergence Drug Research Center, Korea Research Institute of Chemical Technology, Daejeon 34114, Republic of Korea; chemlcj@krict.re.kr; 5 Medicinal Chemistry and Pharmacology, Korea University of Science and Technology (UST), 141 Gajeong-ro, Yuseong-gu, Daejeon 34114, Republic of Korea; 6 Department of Biological Systems Engineering, University of Nebraska-Lincoln, Lincoln, NE 68588, USA; hsong5@unl.edu; 7 Department of Food Science and Technology, Nebraska Food for Health Center, University of Nebraska-Lincoln, Lincoln, NE 68588, USA

**Keywords:** antibacterial, 2-(Amino)quinazolin-4(3*H*)-one, methicillin-resistant *Staphylococcus aureus* (MRSA), multi-antibiotic-resistance, quinazoline

## Abstract

**Background/Objectives:** The rise in methicillin-resistant *Staphylococcus aureus* (MRSA) demands new therapeutic strategies. In this study, a series of 2-(amino)quinazolin-4(3*H*)-one derivatives were synthesized and evaluated for antistaphylococcal activity. **Methods/Results:** Through screening against *S. aureus* ATCC25923 and USA300 JE2, several submicromolar inhibitors were identified. Among them, compound **6l**, which contains a 7-chloro substituent on the key parental scaffold, exhibited strong overall antibacterial activity (MIC_50_: 1.0 µM, ATCC25923; 0.6 µM, JE2) and served as a lead for further structural optimization. Structure–activity relationship analysis showed that substitution at the 2-position was critical, with its optimized analog **6y** (3,4-difluorobenzylamine) exhibiting the highest potency (MIC_50_: 0.36 µM, ATCC25923; 0.02 µM, JE2). Cytotoxicity assays in HepG2 cells revealed six compounds with IC_50_ values above 20 µM, yielding efficacy windows greater than 10. Compound **6y** exhibited an exceptional index (~885). Consistently, in an H460 lung epithelial infection model mimicking MRSA pneumonia, **6y** significantly reduced intracellular bacterial loads with minimal host cell damage, outperforming comparator compounds. **Conclusions:** These findings highlight 2-(amino)quinazolin-4(3*H*)-one derivatives, particularly **6y**, as promising leads for the development of new antistaphylococcal agents.

## 1. Introduction

**Figure 1:**
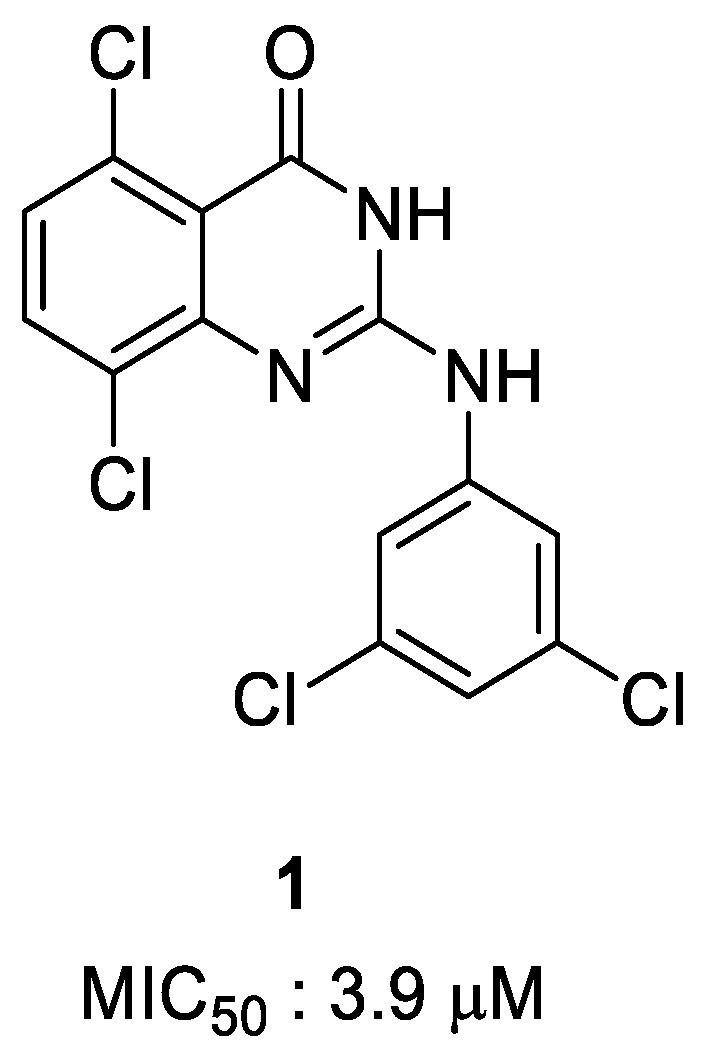
Hit compound against *S. aureus* ATCC25923.

*Staphylococcus aureus* infections, particularly those caused by methicillin-resistant strains (MRSA), remain a major global health challenge [1,2]. MRSA is a significant public health concern in the context of hospital-acquired infections and is associated with the highest age-standardized mortality rate, especially among hospitalized and immunocompromised patients [3,4,5,6,7]. Although MRSA infections are generally treated with vancomycin, daptomycin, clindamycin, or linezolid, the rapid emergence of resistance to these agents threatens their long-term efficacy [8,9]. This escalating resistance crisis highlights the urgent need for novel antibacterial agents with new mechanisms of action and durable therapeutic effectiveness [1,2,10]. In our previous studies, we identified 3-acyl-2-anilino-1,4-dihydroquinolin-4-one derivatives as promising inhibitors of Gram-positive bacteria, including MRSA [11,12,13]. Building upon this work, further screening efforts led to the discovery of 5,8-dichloro-2-((3,5-dichlorophenyl)amino)quinazolin-4(3*H*)-one (compound **1**) as a hit compound, with an MIC_50_ of 3.9 µM against S. aureus ATCC25923 (Figure 1). Quinazolin-4(3*H*)-one scaffolds are structurally versatile and have been widely studied as epidermal growth factor receptor (EGFR) tyrosine inhibitors [14], anti-viral agents [15], and inhibitors of rat lens aldose reductase [16]. Here, we report the design, synthesis, and biological evaluation of a series of 2-(amino)quinazolin-4(3*H*)-one derivatives. Their antistaphylococcal activities were systematically investigated against MRSA strains, and structure–activity relationship (SAR) analyses were performed to identify key determinants of antibacterial potency. The outcomes of this study provide important insights into the development of new quinazolinone-based agents as potential therapeutics against MRSA.

## 2. Results and Discussion

### 2.1. Synthesis of 2-(amino)quinazolin-4(3H)-one Derivatives

**Scheme 1:**
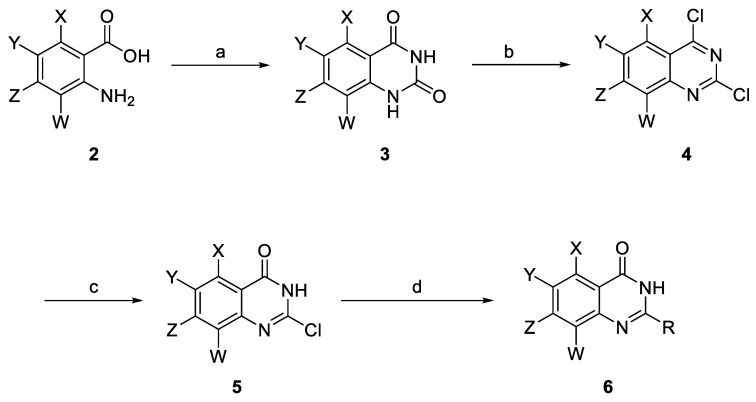
Synthesis of 2-(amino)quinazolin-4(3*H*)-ones derivatives. Reagents and conditions: (a) urea, 160 °C, 20 h, 87%; (b) POCl_3_, TEA, 120 °C, 17 h, 99%; (c) 2 N NaOH, rt, 20 h, 99%; (d) amines, DMF, 85 °C, 16 h, 7–99%.

The synthetic methods used to synthesize 2-(amino)quinazolin-4(3*H*)-one are described in Scheme 1 [15,16]. Anthranilic acids (**2**) underwent urea-mediated cyclocondensation at 160 °C for 20 h to afford quinazolinediones (**3**). Chlorination of quinazolinediones (**3**) with POCl_3_ in the presence of triethylamine under reflux conditions (120 °C, 17 h) furnished dichloroquinazolines (**4**). Subsequent base-promoted hydrolysis at C4 (2 N NaOH, rt, 20 h) provided 2-chloro-4(3*H*)-quinazolinones (**5**). Finally, nucleophilic substitution at C2 with substituted anilines or amines in DMF at 85 °C for 16 h afforded the target 2-(amino)quinazolin-4(3*H*)-ones (**6**).

### 2.2. Antistaphylococcal Activities Screening and SAR Studies

**Figure 2:**
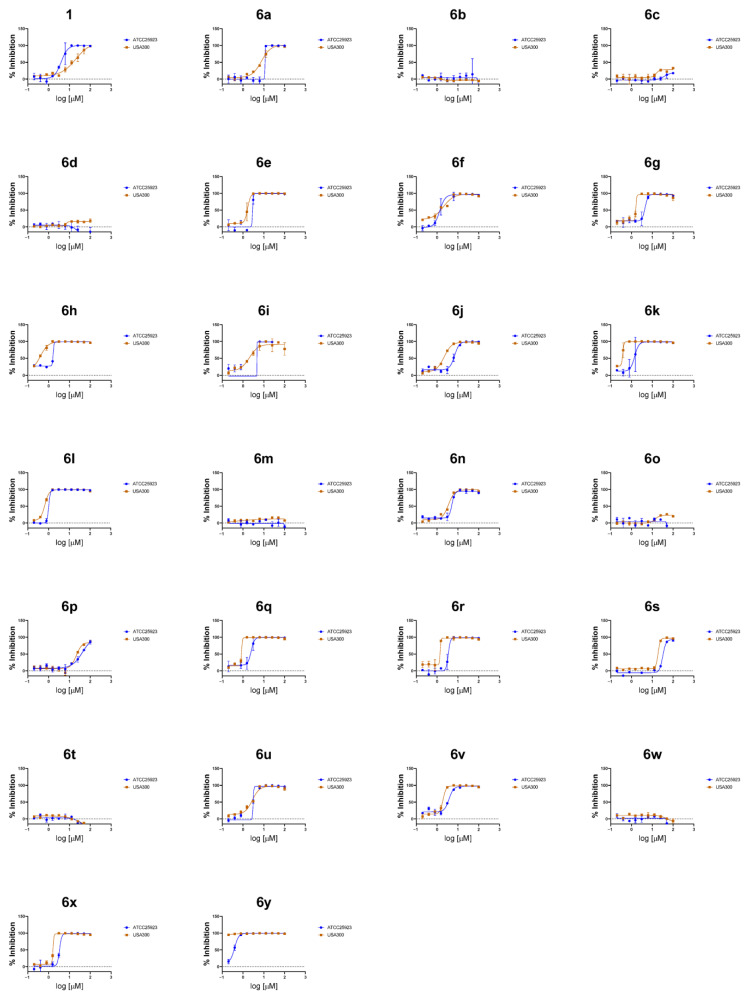
In vitro antibacterial activities of 2-(amino)quinazolin-4(3*H*)-one derivatives*. S. aureus* ATCC25923 (blue) and USA300 JE (orange) strains were incubated with serial dilutions of 2-(amino)quinazolin-4(3*H*)-one derivatives (0.19–100 µM) for 18–24 h. The dose–responses were observed by measuring OD_600nm_. The plots were drawn from each group of strains with nonlinear curve fits.

The antistaphylococcal activities (MIC_50_) of the synthesized compounds were examined via a previously described broth microdilution method against two *S. aureus* strains: ATCC25923, a methicillin-sensitive reference strain, and JE2, a methicillin-resistant strain of the USA300 lineage (Figure 2, Table 1) [17].

Initially, the effects of substituents at positions 5 through 8 in quinazolin-4(3*H*)-one of compound **1**, having been fixed with 3,5-dichloroaniline at position 2, were investigated (**6a**–**l**). The compound with 7,8-dichloro groups (**6a**) exhibited decreased antibacterial activity against ATCC25923 (MIC_50_ = 10.8 μM) but increased activity against JE2 (MIC_50_ = 6.9 μM) compared to compound **1**. A morpholine substitution at position 7 (**6b**) and an electron-donating group of 6,7-dimethoxy (**6c**) or a 7-methoxy group (**6d**) resulted in the complete abolition of antibacterial activity. Compounds with 7-nitro groups (**6e**), 6,8-dibromo groups (**6f**), 6,7-difluoro groups (**6g**), 6,7-dichlro groups (**6h**), and 6,8-dichloro groups (**6i**), as well as a compound with no substituents (**6j**), showed at least 6.5-fold better antibacterial activities (MIC_50_ = 0.3–1.9 μM) against JE2 with similar activities (MIC_50_ = 3.0–6.0 μM) against ATCC25923, compared to compound **1**. Interestingly, 7-trifluoromethyl (**6k**) and 7-chloro (**6l**) substituents resulted in increased antibacterial activity against both strains, showing lower MIC_50_ values (MIC_50_ = 1.3 and 1.0 μM for ATCC25923, MIC_50_ = 0.3 and 0.6 μM for JE2, respectively).

Since the 7-chloro (**6l**) substituent showed the overall best activity, subsequent investigation was conducted to examine the effect of substituents at position 2 in compound **6l** (**6m**–**y**). Decreased antibacterial activities were observed with substituents of 3-methoxyaniline (**6p**) as well as cyclohexylmethylamine (**6s**) at position 2, and the complete loss of activity occurred with substituents of aniline (**6m**), 2,4-difluoroaniline (**6o**), 2-chloroaniline (**6t**), and 3,5-difluorobenzylamine (**6w**) at position 2. Compounds with 3-fluoroaniline (**6n**), 3,5-difluoroaniline (**6q**), 3-trifluoromethylaniline (**6r**), 3-chloroaniline (**6u**), 4-chloroaniline (**6v**), and 2,4-dichlorobenzylamine (**6x**) exhibited less antibacterial activity than compound **61** against both strains (MIC_50_ = 2.1–4.5 μM for ATCC25923, MIC_50_ = 0.9–3.0 μM for JE2). Among all derivatives, compound **6y**, containing a 3,4-difluorobenzylamine moiety at the 2-position, exhibited the highest antibacterial activity, with MIC_50_ values of 0.36 µM for ATCC25923 and 0.02 µM for JE2.

Antistaphylococcal activity tests suggested that the synthesized derivatives were improved for inhibitory effects against MRSA USA300 compared to Compound **1,** except those with no activity for both strains, ATCC25923 and USA300. Compound **6y** demonstrated nanomolar MIC_50_ values against USA300, highlighting its potential as a lead candidate for MRSA treatment.

### 2.3. In Vitro Cytotoxicity

The in vitro cytotoxicity of the compounds was evaluated by measuring the viability of HepG2 cells, a human liver cell line, following treatment in a dose–response manner, as previously described [12]. Fifteen compounds with MIC_50_ values below 5 µM against JE2 were selected for the cytotoxicity testing (Table 1). Among these, six compounds exhibited IC_50_ values exceeding 20 µM, with the highest IC_50_ value observed for compound **6l** (62 µM). The efficacy window was calculated as the ratio of IC_50_ in HepG2 cells to MIC_50_ against JE2. All compounds with IC_50_ values above 20 µM demonstrated efficacy windows greater than 10, indicating a favorable safety profile. Notably, compound **6y** displayed the most promising efficacy window, with an MIC_50 USA300_ of 0.02 µM, approximately 885 times lower than its IC_50 HepG2_ value (20.2 µM).

### 2.4. Antibacterial Activity in a Cell Infection Model

**Figure 3:**
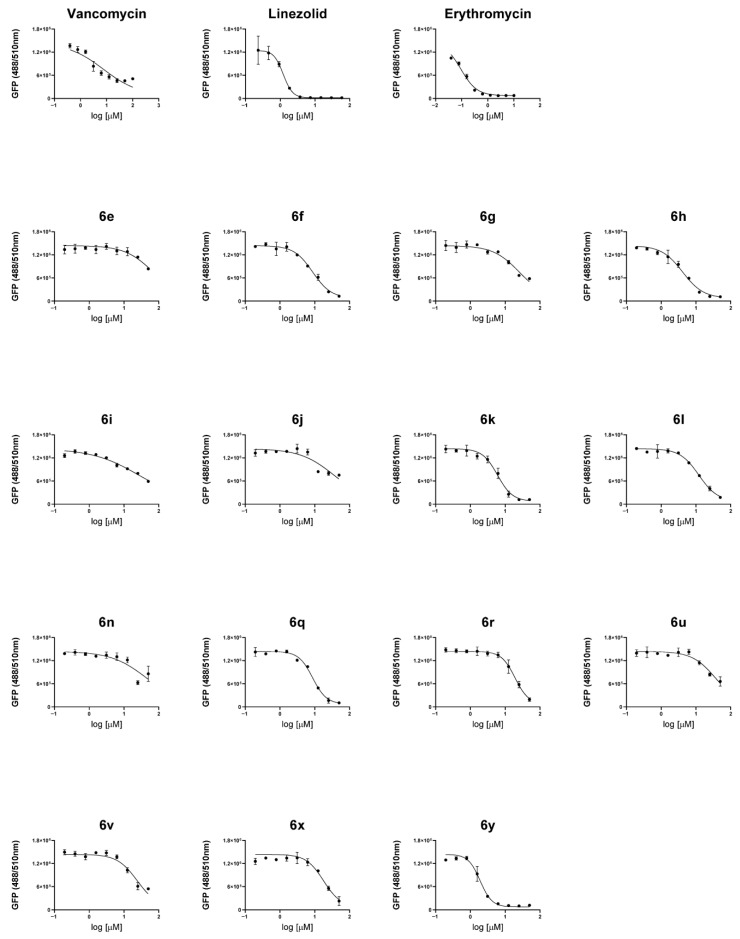
Antibacterial activities of 2-(amino)quinazolin-4(3*H*)-one derivatives in the H460 infection model. H460 cells were infected with *S. aureus* USA300 JE strain expressing GFP, followed by treatment with serial dilutions of 2-(amino)quinazolin-4(3*H*)-one derivatives. After incubation at 37 °C for 24 h with 5% CO_2_, fluorescence signals of the bacteria were detected at 488 nm/510 nm using a multilabel plate reader.

**Figure 4:**
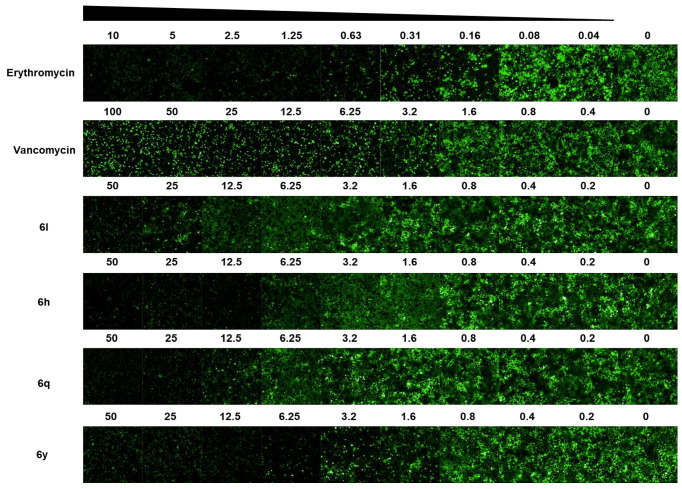
Dose–response images of 2-(amino)quinazolin-4(3*H*)-one derivatives in the H460 infection model. H460 cells were infected with *S. aureus* USA300 JE strain expressing GFP, followed by treatment with serial dilutions of 2-(amino)quinazolin-4(3*H*)-one derivatives **6l**, **6h**, **6q**, and **6y**. After incubation at 37 °C for 24 h with 5% CO_2_, images were obtained by a confocal microscope with a 20× water objective lens detecting fluorescence signals of the bacteria (green) at 488 nm/500–550 nm.

**Figure 5:**
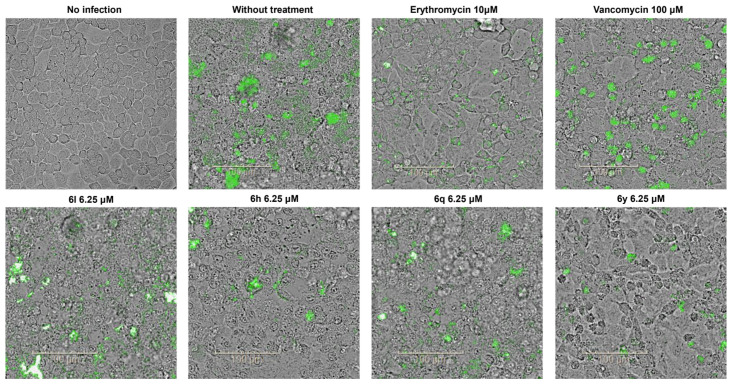
Images of infected H460 cells with treatment using selected 2-(amino)quinazolin-4(3*H*)-one derivatives. H460 cells were infected with *S. aureus* USA300 JE strain expressing GFP, followed by treatment with serial dilutions of 2-(amino)quinazolin-4(3*H*)-one derivatives. After incubation at 37 °C for 24 h with 5% CO_2_, brightfield images of H460 cells, as well as confocal fluorescence images of USA300 (green), were obtained and merged using an automated microlens-enhanced spinning disk confocal microscope.

*Staphylococcus aureus*, including MRSA, can invade host cells and replicate intracellularly, which complicates treatment with current therapies [18,19,20,21,22,23]. To evaluate the intracellular antibacterial activities of the selected compounds, the fifteen compounds tested for cytotoxicity were further assessed using a cell infection model based on H460 human lung epithelial cells, which mimic MRSA-mediated pneumonia. H460 cells were infected with the USA300 JE2 expressing green fluorescent protein (GFP) and subsequently treated with the compounds in a dose–response manner, along with three reference antibiotics: vancomycin, linezolid, and erythromycin. After 24 h of incubation, bacterial loads were quantified by measuring GFP fluorescence in each well. MIC_50_ values determined in the H460 infection model (MIC_50 ex vivo)_ were generally higher than those obtained from the in vitro tests, likely due to either limited compound penetration into host cells or reduced antibacterial effect of the compounds against intracellular bacteria (Figure 3, Table 2). Despite this reduced efficacy in the H460 infection model, four compounds—**6h**, **6l**, **6q**, and **6y**—showed at least a two-fold efficacy window (IC_50 HepG2_/MIC_50 ex vivo_) (Table 2, Figure 4). Notably, compound **6y,** which showed the most potency in vitro, exhibited an MIC_50 ex vivo_ value of 1.9 µM, which was superior to vancomycin (MIC_50 ex vivo_ = 7.7 µM) and similar to linezolid (MIC_50 ex vivo_ = 1.2 µM). The largest efficacy window was also observed for compound **6y** (10.6-fold), which caused relatively minimal distortion of H460 cell morphology while markedly reducing the bacterial load compared with the other compounds at the same concentration of 6.25 µM (Table 2, Figure 5). These findings underscore the potential of compound **6y** as a promising lead for further development as a novel antistaphylococcal agent.

## 3. Materials and Methods

### 3.1. Materials

All reagents and solvents were obtained from commercial suppliers (Sigma-Aldrich, Burlington, MA, USA; Alfa Aesar, Ward Hill, MA, USA; TCI, Tokyo, Japan; Combi-Blocks, San Diego, CA, USA; Angene, Nanjing, China) and used as received unless otherwise noted. Organic solvents were removed under reduced pressure using a rotary evaporator (Rotavapor R-300, Büchi, Flawil, Switzerland). Reaction progress was monitored by thin-layer chromatography (TLC) on a silica gel 60 F254 plate (Merck, Darmstadt, Germany). Crude products were purified by flash column chromatography on silica gel (230–400 mesh, Merck).

Liquid chromatography was performed on a UPLC (Acquity H class, Waters, Milford, MA, USA) using a C18 column (Waters, Acquity UPLC BCH C18, 1.7 μM, 2.1 × 50 mm) maintained at 40 °C. Mobile phase A was water with 0.1% formic acid; mobile phase B was acetonitrile with 0.1% formic acid. The gradient program was as follows: 0–1 min, 5% B; 1–3 min, 5%→95% B (linear); 3–4 min, 95% B (hold); 4–5 min, 95%→5% B; 5–7 min, 5% B (re-equilibration). The flow rate was 0.3 mL/min, and the injection volume was 1 μL. UV detection was carried out with a DAD at 254 nm. Mass spectrometry was performed on an SQ detector 2 (Waters) equipped with an ESI source operating in positive or negative ion mode. Acquisition was carried out in full scan, *m*/*z* 100–1000. Key source parameters were as follows: capillary voltage 1.0 kV; desolvation temperature 350 °C; drying gas 650 L/h; nebulizer 35 psi. Data were processed using MassLynx V4.2. HRMS analysis in EI mode, 70eV, and resolution 5000 was conducted using a JEOL JMS-700 High-Resolution Mass Spectrometer (JEOL Ltd., Tokyo, Japan).

NMR spectra were recorded at 300 MHz, 400 MHz, 500 MHz (^1^H), and 100 MHz, 125 MHz (^13^C), and chemical shifts are reported in δ values downfield from TMS or relative to residual chloroform (7.26 ppm, 77.16 ppm), with residual DMSO (2.50 ppm, 39.52 ppm) as an internal standard. Data are reported in the following manner: chemical shift, multiplicity, coupling constant (*J*) in hertz (Hz), and integrated intensity and assignment (when possible). Multiplicities are reported using the following abbreviations: s, singlet; d, doublet; dd, doublet of doublets; t, triplet; q, quadruplet; m, multiplet; br s, broad signal; app, apparent.

### 3.2. Chemistry

#### 3.2.1. 5,8-Dichloro-2-((3,5-dichlorophenyl)amino)quinazolin-4(3*H*)-one (**1**)

Following the procedure of compound **6l**. The compound **1** (71% chemical yield) was obtained as a white solid. ^1^H NMR (300 MHz, (CD_3_)_2_SO) δ 11.36 (s, 1H), 9.32 (s, 1H), 8.27 (d, *J* = 1.9 Hz, 1H), 8.07 (d, *J* = 1.9 Hz, 2H), 7.80 (d, *J* = 3.5 Hz, 1H), 7.26 (t, *J* = 1.8 Hz, 1H) ppm; ^13^C NMR (100 MHz, (CD_3_)_2_SO) δ 159.31, 148.19, 147.44, 140.82, 134.03, 133.84, 131.54, 127.91, 125.77, 121.68, 117.36, 116.69 ppm; HRMS (EI) *m*/*z*: [M]^+^ calcd for C_14_H_7_C_l4_N_3_O: 372.9343; found: 372.9343.

#### 3.2.2. 7-Chloroquinazoline-2,4(1*H*,3*H*)-dione (**3l**)

4-Chloroanthranilic acid (**2l**, 5 g, 29.1 mmoL) and urea (30 g, 582 mmoL) were poured into a 100 mL round-bottom flask. Then, the reaction mixture was heated at 150 °C for 22 h. After the reaction was completed, the mixture was cooled down to room temperature. Then, 50 mL of water was poured into the reaction mixture and heated at 110 °C for 1 h. The reaction mixture was cooled down using an ice bath, and the white solid was precipitated. The white solid was filtered and washed with water and hexane. The white solid (**3l**, 4.95 g, 87%) was dried in vacuo and used in the next step without further purification. ^1^H NMR (400 MHz, (CD_3_)_2_SO) δ 11.32 (s, 2H), 7.87 (d, *J* = 8.4 Hz, 1H), 7.33–7.06 (m, 2H).

#### 3.2.3. 2,4,7-Trichloroquinazoline (**4l**)

The compound **3l** (5.6 g, 28.5 mmoL) was dissolved in triethylamine (8 mL, 57 mmoL). Then, POCl_3_ (24 mL, 256.4 mmoL) was slowly added to the stirred reaction mixture. The reaction mixture was heated at 115 °C for 15 h. After the reaction was completed, the solvents were evaporated with toluene several times. The residue was diluted with water and extracted with ethyl acetate several times. The extracted organic layer was dried over MgSO_4_ and filtered, concentrated in vacuo. The residue **4l** (6.63 g, 99.6%) was used in the next step without further purification. ^1^H NMR (300 MHz, CDCl_3_) δ 8.22–8.19 (d, *J* = 9.0 Hz, 1H), 8.00 (s, 1H), 7.71–7.68 (d, *J* = 9 Hz, 1H).; LC/MS (ESI) *m*/*z*: [M + H]^+^ calcd for C_8_H_3_Cl_3_N_2_: 232.9; found: 232.9.

#### 3.2.4. 2,7-Dichloroquinazolin-4(3*H*)-one (**5l**)

The compound **4l** (2 g, 8.6 mmoL) was dissolved in 2 N NaOH solution (13 mL, 26 mmoL) and stirred at room temperature for 12 h. After the reaction was completed, the acetic acid (1.5 mL, 26 mmoL) was added to the reaction mixture. The aqueous phase was extracted with ethyl acetate several times. The extracted organic layer was dried over MgSO_4_ and filtered, concentrated in vacuo. The residue **5l** (1.8 g, 99%) was used in next step without further purification. ^1^H NMR (300 MHz, (CD_3_)_2_CO) δ 8.14 (d, *J* = 8.5 Hz, 1H), 7.63 (d, *J* = 2.1 Hz, 1H), 7.56 (dd, *J* = 8.5, 2.1 Hz, 1H).; LC/MS (ESI) *m*/*z*: [M + H]^+^ calcd for C_8_H_4_Cl_2_N_2_O: 215.0; found: 215.1.

#### 3.2.5. 7,8-Dichloro-2-((3,5-dichlorophenyl)amino)quinazolin-4(3*H*)-one (**6a**)

Following the procedure of compound **6l**. Compound **6a** (10% chemical yield) was obtained as a white solid. mp > 300 °C; ^1^H NMR (500 MHz, DMSO) δ 11.05 (s, 1H), 9.07 (s, 1H), 8.00 (d, *J* = 1.9 Hz, 2H), 7.93 (d, *J* = 8.5 Hz, 1H), 7.43 (d, *J* = 8.5 Hz, 1H), 7.19 (d, *J* = 2.2 Hz, 1H).; ^13^C NMR (125 MHz, DMSO) δ 160.26, 147.73, 140.53, 137.25, 133.71, 124.90, 123.90, 121.49, 118.17, 117.43.; HRMS (EI) *m*/*z*: [M]^+^ calcd for C_14_H_7_C_l4_N_3_O: 372.9343; found: 372.9340.

#### 3.2.6. 2-((3,5-Dichlorophenyl)amino)-7-morpholinoquinazolin-4(3*H*)-one (**6b**)

The 7-Chloro-2-((3,5-dichlorophenyl)amino)quinazolin-4(3*H*)-one (**6l**, 50 mg, 0.15 mmol) was dissolved in morpholine (1 mL). The test tube was sealed with a cap and heated at 130 °C for 22 h. After the reaction was completed, ice water was poured into the reaction mixture, and a precipitate formed. The precipitate was filtered and washed with water and hexane thoroughly. The solid was dried in a vacuum, and **6b** (11 mg, 19% yield) was obtained as a white solid. ^1^H NMR (300 MHz, (CD_3_)_2_SO) δ 9.02 (s, 1H), 7.82 (s, 3H), 7.78 (s, 1H), 7.21 (t, *J* = 1.9 Hz, 1H), 6.97 (d, *J* = 9.2 Hz, 1H), 6.73 (d, *J* = 2.4 Hz, 1H), 3.73 (d, *J* = 4.9 Hz, 4H), 3.30 (d, *J* = 6.5 Hz, 4H).; LC/MS (ESI) *m*/*z*: [M + H]^+^ calcd for C_18_H_16_Cl_2_N_4_O_2_: 391.1; found: 391.4.

#### 3.2.7. 2-((3,5-Dichlorophenyl)amino)-6,7-dimethoxyquinazolin-4(3*H*)-one (**6c**)

Following the procedure of compound **6l**. Compound **6c** (90% chemical yield) was obtained as a gray solid. mp 277–279 °C; ^1^H NMR (400 MHz, (CD_3_)_2_SO) δ 11.52 (s, 1H), 11.01 (s, 1H), 7.87 (d, *J* = 1.9 Hz, 2H), 7.34 (s, 1H), 7.16 (t, *J* = 1.9 Hz, 1H), 6.91 (s, 1H), 3.90 (s, 3H), 3.82 (s, 3H).; ^13^C NMR (100 MHz, (CD_3_)_2_SO) δ 154.82, 146.45, 142.12, 134.09, 120.70, 116.19, 111.05, 105.60, 55.88, 55.62.; HRMS (EI) *m*/*z*: [M]^+^ calcd for C_16_H_13_Cl_2_N_3_O_3_: 365.0334; found: 365.0334.

#### 3.2.8. 2-((3,5-Dichlorophenyl)amino)-7-methoxyquinazolin-4(3*H*)-one (**6d**)

Following the procedure of compound **6l**. Compound **6d** (91% chemical yield) was obtained as a white solid. ^1^H NMR (400 MHz, (CD_3_)_2_SO) δ 10.89 (s, 1H), 8.98 (s, 1H), 7.90–7.86 (m, 1H), 7.84–7.79 (m, 2H), 7.23 (t, *J* = 1.9 Hz, 1H), 6.88–6.85 (m, 2H), 3.88 (s, 3H) ppm; LC/MS (ESI) *m*/*z*: [M + H]^+^ calcd for C_15_H_11_Cl_2_N_3_O_2_: 336.0; found: 336.3.

#### 3.2.9. 2-((3,5-Dichlorophenyl)amino)-7-nitroquinazolin-4(3*H*)-one (**6e**)

Following the procedure of compound **6l**. Compound **6e** (40% chemical yield) was obtained as a yellow solid. ^1^H NMR (400 MHz, (CD_3_)_2_SO) δ 11.50 (s, 1H), 9.24 (s, 1H), 8.18 (d, *J* = 8.7 Hz, 1H), 8.10 (d, *J* = 2.3 Hz, 1H), 7.98 (dd, *J* = 8.6, 2.3 Hz, 1H), 7.83 (s, 2H), 7.27 (t, *J* = 1.8 Hz, 1H).; ^13^C NMR (100 MHz, DMSO) δ 162.33, 151.41, 140.96, 134.10, 128.08, 122.10, 120.22, 117.97, 117.12.; HRMS (EI) *m*/*z*: [M]^+^ calcd for C_14_H_8_Cl_2_N_4_O_3_: 349.9973; found: 349.9951.

#### 3.2.10. 6,8-Dibromo-2-((3,5-dichlorophenyl)amino)quinazolin-4(3*H*)-one (**6f**)

Following the procedure of compound **6l**. Compound **6f** (10% chemical yield) was obtained as a brown solid. ^1^H NMR (400 MHz, (CD_3_)_2_SO) δ 11.64 (s, 1H), 9.57 (s, 1H), 8.18 (d, *J* = 2.2 Hz, 1H), 8.06 (d, *J* = 1.9 Hz, 2H), 8.01 (d, *J* = 2.3 Hz, 1H), 7.21 (d, *J* = 2.1 Hz, 1H).; ^13^C NMR (100 MHz, (CD_3_)_2_SO) δ 160.73, 148.02, 146.49, 141.06, 139.15, 134.05, 127.75, 121.58, 121.06, 120.89, 117.42, 114.88.; HRMS (EI) *m*/*z*: [M]^+^ calcd for C_14_H_7_Br_2_Cl_2_N_3_O: 460.8333; found: 460.8310.

#### 3.2.11. 2-((3,5-Dichlorophenyl)amino)-6,7-difluoroquinazolin-4(3*H*)-one (**6g**)

Following the procedure of compound **6l**. Compound **6g** (11% chemical yield) was obtained as a brown solid.; mp > 300 °C; ^1^H NMR (400 MHz, (CD_3_)_2_SO) δ 10.57 (s, 1H), 8.33 (s, 1H), 7.65 (d, *J* = 1.9 Hz, 2H), 7.36–7.21 (m, 2H).; ^13^C NMR (100 MHz, (CD_3_)_2_SO) δ 162.78, 160.44, 141.24, 140.46, 134.72, 134.22, 122.93, 122.53, 117.37, 115.47.; HRMS (EI) *m*/*z*: [M]^+^ calcd for C_14_H_7_Cl_2_F_2_N_3_O: 340.9934; found: 340.9922.

#### 3.2.12. 6,7-Dichloro-2-((3,5-dichlorophenyl)amino)quinazolin-4(3*H*)-one (**6h**)

Following the procedure of compound **6l**. Compound **6h** (7% chemical yield) was obtained as a white solid. ^1^H NMR (300 MHz, (CD_3_)_2_SO) δ 11.38 (s, 1H), 9.21 (s, 1H), 8.05 (s, 1H), 7.79 (s, 2H), 7.69 (s, 1H), 7.25 (s, 1H).; LC/MS (ESI) *m*/*z*: [M + H]^+^ calcd for C_14_H_7_Cl_4_N_3_O: 373.9; found: 374.4.

#### 3.2.13. 6,8-Dichloro-2-((3,5-dichlorophenyl)amino)quinazolin-4(3*H*)-one (**6i**)

Following the procedure of compound **6l**. Compound **6i** (12% chemical yield) was obtained as a white solid. mp > 300 °C; ^1^H NMR (400 MHz, (CD_3_)_2_SO) δ 11.41 (s, 1H), 9.43 (s, 1H), 7.99 (s, 2H), 7.97–7.89 (m, 1H), 7.89–7.78 (m, 1H), 7.21 (s, 1H).; ^13^C NMR (100 MHz, (CD_3_)_2_SO) δ 160.62, 148.05, 145.51, 141.47, 134.53, 134.31, 130.39, 127.51, 124.59, 122.16, 121.26, 117.81.; HRMS (EI) *m*/*z*: [M]^+^ calcd for C_14_H_7_Cl_4_N_3_O: 372.9343; found: 372.9337.

#### 3.2.14. 2-((3,5-Dichlorophenyl)amino)quinazolin-4(3*H*)-one (**6j**)

Following the procedure of compound **6l**. Compound **6j** (80% chemical yield) was obtained as a white solid.; mp > 300 °C; ^1^H NMR (400 MHz, (CD_3_)_2_SO) δ 11.08 (s, 1H), 9.04 (s, 1H), 7.98 (dd, *J* = 7.9, 1.6 Hz, 1H), 7.81 (d, *J* = 2.0 Hz, 2H), 7.67 (ddd, *J* = 8.5, 7.1, 1.6 Hz, 1H), 7.42 (d, *J* = 8.1 Hz, 1H), 7.30–7.25 (m, 1H), 7.19 (t, *J* = 1.9 Hz, 1H).; ^13^C NMR (100 MHz, (CD_3_)_2_SO) δ 162.17, 147.44, 142.03, 135.02, 134.48, 126.43, 125.76, 124.21, 121.88, 119.07, 117.87.; HRMS (EI) *m*/*z*: [M]^+^ calcd for C_14_H_9_Cl_2_N_3_O: 305.0123; found: 305.0127.

#### 3.2.15. 2-((3,5-Dichlorophenyl)amino)-7-(trifluoromethyl)quinazolin-4(3*H*)-one (**6k**)

Following the procedure of compound **6l**. Compound **6k** (33% chemical yield) was obtained as a pink solid.; mp > 300 °C; ^1^H NMR (400 MHz, (CD_3_)_2_SO) δ 11.29 (s, 1H), 9.10 (s, 1H), 8.12 (d, *J* = 8.2 Hz, 1H), 7.78 (s, 2H), 7.66 (s, 1H), 7.54–7.48 (m, 1H), 7.20 (d, *J* = 2.0 Hz, 1H).; ^13^C NMR (100 MHz, (CD_3_)_2_SO) δ 160.81, 149.50, 148.01, 141.03, 134.03, 127.57, 125.00, 122.29, 121.87, 121.42, 119.18, 117.71; HRMS (EI) *m*/*z*: [M]^+^ calcd for C_15_H_8_Cl_2_F_3_N_3_O: 372.9997; found: 372.9996.

#### 3.2.16. 7-Chloro-2-((3,5-dichlorophenyl)amino)quinazolin-4(3*H*)-one (**6l**)

To a stirred solution of compound **5l** (200 mg, 0.93 mmoL) in DMF (3.1 mL), 3,5-dichloroaniline (**7l**, 452 mg, 2.8 mmoL) was slowly added. The reaction mixture was heated at 85 °C for 19 h. The reaction mixture was cooled down to room temperature, and precipitates were formed. The precipitates were filtered and washed with water and hexane thoroughly. The white solid (285 mg, 90%) was dried in vacuo. If the precipitates were not formed, the reaction mixture was purified by prep HPLC (Shim-pack PREP-ODS, H_2_O:CH_3_CN = 90:10 to H_2_O:CH_3_CN = 10:90, flow rate = 12 mL/min, 40 °C, λ = 254 nm, retention time: 30 min).; mp > 300 °C; ^1^H NMR (400 MHz, (CD_3_)_2_SO) δ 11.09 (s, 1H), 9.02 (s, 1H), 7.93 (d, *J* = 8.4 Hz, 1H), 7.76 (s, 2H), 7.41 (s, 1H), 7.32–7.05 (m, 2H); ^13^C NMR (100 MHz, (CD_3_)_2_SO) δ 160.88, 150.47, 147.85, 141.09, 139.13, 134.00, 127.82, 124.47, 123.77, 121.71, 117.58; HRMS (FAB) *m*/*z*: [M + H]^+^ calcd for C_14_H_8_Cl_3_N_3_O: 339.9733; found: 339.9813.

#### 3.2.17. 7-Chloro-2-(phenylamino)quinazolin-4(3*H*)-one (**6m**)

Following the procedure of compound **6l**. Compound **6m** (94% chemical yield) was obtained as a white solid. mp 258–261 °C; ^1^H NMR (400 MHz, (CD_3_)_2_SO) δ 10.90 (s, 1H), 8.75 (s, 1H), 7.94 (d, *J* = 8.4 Hz, 1H), 7.76–7.68 (m, 2H), 7.43 (d, *J* = 2.0 Hz, 1H), 7.38–7.32 (m, 2H), 7.23 (dd, *J* = 8.4, 2.1 Hz, 1H), 7.09–7.02 (m, 1H).; ^13^C NMR (100 MHz, (CD_3_)_2_SO) δ 160.99, 151.31, 148.39, 139.09, 138.63, 128.87, 127.88, 124.39, 123.15, 122.84, 119.62, 117.17.; HRMS (EI) *m*/*z*: [M]^+^ calcd for C_14_H_10_ClN_3_O: 271.0512; found: 271.0531.

#### 3.2.18. 7-Chloro-2-((3-fluorophenyl)amino)quinazolin-4(3*H*)-one (**6n**)

Following the procedure of compound **6l**. Compound **6n** (93% chemical yield) was obtained as a white solid. mp 271–273 °C; ^1^H NMR (400 MHz, (CD_3_)_2_SO) δ 10.95 (s, 1H), 8.96 (s, 1H), 7.94 (d, *J* = 8.4 Hz, 1H), 7.91–7.81 (m, 1H), 7.48 (d, *J* = 2.0 Hz, 1H), 7.39–7.29 (m, 2H), 7.25 (dd, *J* = 8.5, 2.1 Hz, 1H), 6.86 (td, *J* = 8.7, 2.3 Hz, 1H).; ^13^C NMR (100 MHz, (CD_3_)_2_SO) δ 163.51, 161.01 (d, *J*_(C-F)_ = 21.0 Hz), 150.93, 148.11, 140.45 (d, *J*_(C-F)_ = 11.4 Hz), 139.16, 130.34 (d, *J*_(C-F)_ = 9.8 Hz), 127.86, 124.55, 123.52, 117.32, 115.23, 109.09 (d, *J*_(C-F)_ = 21.2 Hz), 106.32 (d, *J*_(C-F)_ = 27.0 Hz).; HRMS (EI) *m*/*z*: [M]^+^ calcd for C_14_H_9_ClFN_3_O: 289.0418; found: 289.0408.

#### 3.2.19. 7-Chloro-2-((2,4-difluorophenyl)amino)quinazolin-4(3*H*)-one (**6o**)

Following the procedure of compound **6l**. Compound **6o** (99% chemical yield) was obtained as a white solid. mp 288–290 °C; ^1^H NMR (400 MHz, (CD_3_)_2_SO) δ 11.20 (s, 1H), 8.59 (s, 1H), 8.45 (td, *J* = 9.2, 5.9 Hz, 1H), 7.92 (d, *J* = 8.5 Hz, 1H), 7.39–7.31 (m, 2H), 7.22 (dd, *J* = 8.5, 2.1 Hz, 1H), 7.08 (tdd, *J* = 8.4, 3.0, 1.5 Hz, 1H).; ^13^C NMR (100 MHz, (CD_3_)_2_SO) δ 160.87, 158.76 (d, *J*_(C-F)_ = 12.0 Hz), 156.34 (d, *J*_(C-F)_ = 11.7 Hz), 154.30 (d, *J*_(C-F)_ = 12.4 Hz), 151.85 (d, *J*_(C-F)_ = 12.5 Hz), 150.95, 148.49, 139.10, 127.88, 124.38, 123.68 (d, *J*_(C-F)_ = 9.1 Hz), 123.37, 117.23, 111.10 (dd, *J*_(C-F)_ = 21.6, 3.6 Hz), 104.74–102.78 (m). HRMS (EI) *m*/*z*: [M]^+^ calcd for C_14_H_8_ClF_2_N_3_O: 307.0324; found: 307.0327.

#### 3.2.20. 7-Chloro-2-((3-methoxyphenyl)amino)quinazolin-4(3*H*)-one (**6p**)

Following the procedure of compound **6l**. Compound **6p** (99% chemical yield) was obtained as a white solid. mp 242–245 °C; ^1^H NMR (400 MHz, (CD_3_)_2_SO) δ 10.87 (s, 1H), 8.76 (s, 1H), 7.94 (d, *J* = 8.4 Hz, 1H), 7.54 (d, *J* = 2.3 Hz, 1H), 7.43 (d, *J* = 2.0 Hz, 1H), 7.27–7.19 (m, 2H), 7.17–7.09 (m, 1H), 6.63 (dd, *J* = 8.1, 2.5 Hz, 1H), 3.78 (s, 3H).; ^13^C NMR (100 MHz, (CD_3_)_2_SO) δ 160.94, 159.67, 151.20, 148.29, 139.80, 139.12, 129.61, 127.87, 124.42, 123.25, 117.19, 111.76, 108.31, 105.29, 55.05.; HRMS (EI) *m*/*z*: [M]^+^ calcd for C_15_H_12_ClN_3_O_2_: 301.0618; found: 301.0603.

#### 3.2.21. 7-Chloro-2-((3,5-difluorophenyl)amino)quinazolin-4(3*H*)-one (**6q**)

Following the procedure of compound **6l**. C **6q** (88% chemical yield) was obtained as a yellow solid. mp > 300 °C; ^1^H NMR (400 MHz, (CD_3_)_2_SO) δ 11.04 (s, 1H), 9.10 (s, 1H), 7.93 (d, *J* = 8.4 Hz, 1H), 7.56–7.36 (m, 3H), 7.25 (dd, *J* = 8.5, 2.2 Hz, 1H), 6.84 (tt, *J* = 9.3, 2.4 Hz, 1H).; ^13^C NMR (100 MHz, (CD_3_)_2_SO) δ 163.70 (d, *J*_(C-F)_ = 15.6 Hz), 161.29 (d, *J*_(C-F)_ = 15.5 Hz), 160.90, 150.59, 147.89, 141.31 (t, *J*_(C-F)_ = 14.0 Hz), 139.18, 127.82, 124.66, 123.81, 117.44, 102.27 (d, *J*_(C-F)_ = 29.6 Hz), 97.60 (t, *J*_(C-F)_ = 26.2 Hz).; HRMS (EI) *m*/*z*: [M]^+^ calcd for C_14_H_8_ClF_2_N_3_O: 307.0324; found: 307.0328.

#### 3.2.22. 7-Chloro-2-((3-(trifluoromethyl)phenyl)amino)quinazolin-4(3*H*)-one (**6r**)

Following the procedure of compound **6l**. Compound **6r** (85% chemical yield) was obtained as a white solid. mp 255–257 °C; ^1^H NMR (400 MHz, (CD_3_)_2_SO) δ 11.09 (s, 1H), 9.09 (s, 1H), 8.24 (s, 1H), 7.96 (d, *J* = 8.4 Hz, 1H), 7.92–7.86 (m, 1H), 7.57 (t, *J* = 8.0 Hz, 1H), 7.42–7.35 (m, 2H), 7.27 (dd, *J* = 8.5, 2.0 Hz, 1H).; ^13^C NMR (100 MHz, (CD_3_)_2_SO) δ 161.00, 150.82, 148.26, 139.52, 139.17, 129.98, 129.69, 129.37, 127.94, 125.53, 124.39, 123.61, 123.30, 122.82, 119.03, 117.41, 115.70 (d, *J*_(C-F)_ = 3.9 Hz).; HRMS (EI) *m*/*z*: [M]^+^ calcd for C_15_H_9_ClFN_3_O: 339.0386; found: 339.0384.

#### 3.2.23. 7-Chloro-2-((cyclohexylmethyl)amino)quinazolin-4(3*H*)-one (**6s**)

Following the procedure of compound **6l**. Compound **6s** (18% chemical yield) was obtained as a white solid. ^1^H NMR (400 MHz, (CD_3_)_2_SO) δ 10.84 (s, 1H), 7.84 (d, *J* = 8.4 Hz, 1H), 7.24 (d, *J* = 2.1 Hz, 1H), 7.08 (dd, *J* = 8.4, 2.1 Hz, 1H), 6.45 (s, 1H), 3.17 (t, *J* = 6.2 Hz, 2H), 1.69 (ddd, *J* = 13.1, 8.5, 4.6 Hz, 4H), 1.62 (t, *J* = 5.2 Hz, 1H), 1.51 (ddt, *J* = 11.1, 7.2, 3.6 Hz, 1H), 1.22–1.12 (m, 3H), 0.95 (td, *J* = 11.7, 3.0 Hz, 2H).; ^13^C NMR (100 MHz, (CD_3_)_2_SO) δ 161.27, 160.93, 151.63, 138.83, 127.94, 123.52, 121.56, 116.09, 46.25, 37.16, 30.29, 26.04, 25.37.; HRMS (EI) *m*/*z*: [M]^+^ calcd for C_15_H_18_ClN_3_O_2_: 291.1138; found: 291.1118.

#### 3.2.24. 7-Chloro-2-((2-chlorophenyl)amino)quinazolin-4(3*H*)-one (**6t**)

Following the procedure of compound **6l**. Compound **6t** (94% chemical yield) was obtained as a white solid. mp > 300 °C; ^1^H NMR (400 MHz, (CD_3_)_2_SO) δ 11.65 (s, 1H), 8.52 (d, *J* = 8.3 Hz, 1H), 8.39 (s, 1H), 7.96 (d, *J* = 8.4 Hz, 1H), 7.52 (dd, *J* = 8.0, 1.5 Hz, 1H), 7.44 (d, *J* = 2.1 Hz, 1H), 7.38 (ddd, *J* = 8.5, 7.4, 1.6 Hz, 1H), 7.27 (dd, *J* = 8.5, 2.1 Hz, 1H), 7.14 (td, *J* = 7.7, 1.6 Hz, 1H).; ^13^C NMR (100 MHz, (CD_3_)_2_SO) δ 161.01, 150.88, 148.44, 139.10, 134.98, 129.34, 127.88, 127.66, 124.49, 123.55, 123.46, 123.15, 117.41.; HRMS (EI) *m*/*z*: [M]^+^ calcd for C_14_H_9_Cl_2_N_3_O: 305.0123; found: 305.0121.

#### 3.2.25. 7-Chloro-2-((3-chlorophenyl)amino)quinazolin-4(3*H*)-one (**6u**)

Following the procedure of compound **6l**. Compound **6u** (99% chemical yield) was obtained as a white solid. mp 284–287 °C; ^1^H NMR (400 MHz, (CD_3_)_2_SO) δ 10.99 (s, 1H), 8.93 (s, 1H), 7.97 (t, *J* = 2.2 Hz, 1H), 7.94 (d, *J* = 8.4 Hz, 1H), 7.53 (dd, *J* = 8.2, 2.2 Hz, 1H), 7.44 (d, *J* = 2.0 Hz, 1H), 7.35 (t, *J* = 8.1 Hz, 1H), 7.25 (dd, *J* = 8.4, 2.1 Hz, 1H), 7.10 (dd, *J* = 8.0, 2.1 Hz, 1H).; ^13^C NMR (100 MHz, (CD_3_)_2_SO) δ 160.94, 160.01, 150.89, 148.15, 140.17, 139.14, 133.20, 130.62, 130.42, 127.90, 124.45, 123.53, 123.37, 122.43, 118.97, 118.70, 118.06, 117.54, 117.33.; HRMS (EI) *m*/*z*: [M]^+^ calcd for C_14_H_9_Cl_2_N_3_O: 305.0123; found: 305.0107.

#### 3.2.26. 7-Chloro-2-((4-chlorophenyl)amino)quinazolin-4(3*H*)-one (**6v**)

Following the procedure of compound **6l**. Compound **6v** (89% chemical yield) was obtained as a white solid. mp > 300 °C; ^1^H NMR (400 MHz, (CD_3_)_2_SO) δ 10.95 (s, 1H), 8.88 (s, 1H), 7.94 (d, *J* = 8.4 Hz, 1H), 7.75 (d, *J* = 8.7 Hz, 2H), 7.43 (d, *J* = 2.1 Hz, 1H), 7.40–7.35 (m, 2H), 7.24 (dd, *J* = 8.4, 2.1 Hz, 1H).; ^13^C NMR (100 MHz, (CD_3_)_2_SO) δ 160.96, 151.06, 148.24, 139.10, 137.64, 128.67, 127.92, 126.40, 124.41, 123.37, 121.24, 117.25.; HRMS (EI) *m*/*z*: [M]^+^ calcd for C_14_H_9_Cl_2_N_3_O: 305.0123; found: 305.0146.

#### 3.2.27. 7-Chloro-2-((3,5-difluorobenzyl)amino)quinazolin-4(3*H*)-one (**6w**)

Following the procedure of compound **6l**. Compound **6w** (49% chemical yield) was obtained as a white solid. mp 253–255 °C; ^1^H NMR (400 MHz, (CD_3_)_2_SO) δ 7.88 (d, *J* = 8.5 Hz, 1H), 7.27 (d, *J* = 2.0 Hz, 1H), 7.15 (dd, *J* = 8.5, 2.1 Hz, 1H), 7.11 (td, *J* = 7.5, 2.9 Hz, 3H), 4.59 (d, *J* = 5.2 Hz, 2H).; ^13^C NMR (100 MHz, (CD_3_)_2_SO) δ 163.62 (d, *J*_(C-F)_ = 13.1 Hz), 161.44, 161.18 (d, *J*_(C-F)_ = 13.2 Hz), 151.43, 144.09 (t, *J*_(C-F)_ = 8.9 Hz), 138.96, 128.06, 122.33, 116.33, 110.79–109.58 (m), 102.35 (t, *J*_(C-F)_ = 25.8 Hz), 42.99. HRMS (EI) *m*/*z*: [M]^+^ calcd for C_15_H_10_ClF_2_N_3_O: 321.0480; found: 321.0480.

#### 3.2.28. 7-Chloro-2-((2,4-dichlorobenzyl)amino)quinazolin-4(3*H*)-one (**6x**)

Following the procedure of compound **6l**. Compound **6x** (10% chemical yield) was obtained as a white solid. ^1^H NMR (300 MHz, (CD_3_)_2_SO) δ 11.23 (s, 1H), 7.88 (d, *J* = 8.5 Hz, 1H), 7.64 (d, *J* = 2.0 Hz, 1H), 7.49–7.39 (m, 2H), 7.26 (d, *J* = 2.0 Hz, 1H), 7.14 (dd, *J* = 8.4, 2.1 Hz, 1H), 7.01 (s, 1H), 4.60 (d, *J* = 5.9 Hz, 2H).; LC/MS (ESI) *m*/*z*: [M + H]^+^ calcd for C_15_H_10_Cl_3_N_3_O: 354.0; found: 353.3.

#### 3.2.29. 7-Chloro-2-((3,4-difluorobenzyl)amino)quinazolin-4(3*H*)-one (**6y**)

Following the procedure of compound **6l**. Compound **6y** (69% chemical yield) was obtained as a yellow solid. ^1^H NMR (400 MHz, (CD_3_)_2_SO) δ 7.89 (d, *J* = 8.4 Hz, 1H), 7.50–7.44 (m, 1H), 7.45–7.33 (m, 2H), 7.31 (d, *J* = 2.0 Hz, 1H), 7.26–7.21 (m, 1H), 7.18 (dd, *J* = 8.5, 2.1 Hz, 1H), 4.56 (d, *J* = 4.4 Hz, 2H).; ^13^C NMR (100 MHz, (CD_3_)_2_SO) δ 161.24, 151.32, 150.52 (d, *J*_(C-F)_ = 12.8 Hz), 149.76 (d, *J*_(C-F)_ = 12.5 Hz), 148.08 (d, *J*_(C-F)_ = 12.7 Hz), 147.33 (d, *J*_(C-F)_ = 12.6 Hz), 139.09, 136.64, 128.14, 124.19 (dd, *J*_(C-F)_ = 6.5, 3.4 Hz), 122.57, 117.37 (d, *J*_(C-F)_ = 17.0 Hz), 116.49 (d, *J*_(C-F)_ = 17.3 Hz), 116.12, 42.83.; HRMS (EI) *m*/*z*: [M]^+^ calcd for C_15_H_10_ClF_2_N_3_O: 321.0480; found: 321.0476.

### 3.3. Biological Studies

#### 3.3.1. Strains and Culture Conditions

*Staphylococcus aureus* USA300, a methicillin-resistant strain, was obtained from the Nebraska Transposon Mutant Library. *Staphylococcus aureus* ATCC25923, a pan-drug-susceptible strain, was purchased from the American Type Culture Collection (ATCC) (Manassas, VA, USA). *S. aureus* strains were grown in Cation-Adjusted Mueller–Hinton broth (CAMHB) (Difco) at 37 °C. Bacterial strain stocks were stored in vials with 25% glycerol at −80 °C. For each experiment, overnight cultures were prepared by inoculating a frozen bacterial strain stock in 10 mL of CAMHB medium and incubating at 37 °C in a shaking incubator (180 rpm). For the preliminary screen and dose–response experiments, 100 µL of overnight culture was inoculated into 10 mL of fresh CAMHB and incubated for 3 h at 37 °C and 180 rpm.

#### 3.3.2. Dose–Response Curve Assay

Antibiotic susceptibility assay was tested according to modified Clinical and Laboratory Standards Institute (CLSI) laboratory guidelines for broth microdilution assays. *S. aureus* was cultured in CAMHB, and bacterial cells were prepared with an OD600 of 0.005. DRC plates were prepared by adding 40 µL of bacterial suspension to each well, where 10 µL of each compound had been dispensed for 17 points of two-fold serial dilutions starting at 40 µM. After incubation of the plates at 37 °C for 18–24 h with 5% CO_2_, the growth of *S. aureus* was detected by measuring the OD600nm using an Ensight (Revvity) spectrometer (Revvity, Waltham, MA, USA).

#### 3.3.3. Cytotoxicity Test of Compounds

To test cytotoxicity with compounds, the human liver cell line HepG2 (ATCC #HB-8065) was used for the assay. HepG2 was cultured in DMEM with 10% fetal bovine serum (FBS) in a 96-well microplate at 37 °C and 5% CO_2_. HepG2 cells were seeded at a final density of 5 × 10^4^ cells/well, and DMEM-containing diluted compounds were added and incubated on the plates at 37 °C in 5% CO_2_ for 24 h. After that, resazurin (final concentration, 0.1 mg/mL) was added to each well and incubated on the plates at 37 °C in 5% CO_2_ for 2 h. To detect the fluorescence at 530 nm excitation and 590 nm emission, an Ensight multimode plate reader was used (Revvity, Waltham, MA, USA).

#### 3.3.4. Effect of Compounds on a Cell Infection Model of *S. aureus*

To see the ex vivo antimicrobial activity of the compound, the human lung carcinoma cell line H460 (ATCC #HTB-177) was used. The H460 cells were cultured in RPMI 1640 medium with 10% fetal bovine serum (FBS) and in a 96-well microplate at 37 °C and 5% CO_2_. Cell lines were cultured for 2 days, until they reached full confluence in the well. *S. aureus* (USA300-containing and pCM29-encoding GFP) was cultured in CAMHB with 10 µg/mL of chloramphenicol, and bacterial cells were harvested. After washing the bacterial cells with phosphate-buffered saline (PBS) and suspending them in RPMI, infected H460 cells with *S. aureus* (1 × 10^7^ CFU) and incubated them for 2.5 h. Samples were washed three times with PBS. Then, cells were treated with gentamicin (50 µg/mL of gentamicin in RPMI) and incubated for 1 h. Samples were washed with PBS, RPMI-containing diluted compounds were added, and then they were incubated at 37 °C in 5% CO_2_ for 24 h. To detect the Green fluorescent protein (GFP) signal, an Ensight multimode plate reader and Opera Phenix Plus (Revvity, Waltham, MA, USA) were used.

#### 3.3.5. Confocal Images

Ex vivo infection model images were generated using the Opera Phenix Plus high-content imaging system. Images were obtained by a confocal microscope with a 20× water objective lens. To detect the bacteria fluorescence signal (GFP), the Alexa 488 channel (488 nm laser/500–550 filter, Thermo Fisher Scientific, Waltham, MA, USA) was used, and the brightfield channel was used for H460 cell images.

## 4. Conclusions

This study identified and optimized 2-(amino)quinazolin-4(3*H*)-one derivatives as promising antistaphylococcal agents. Compound **6l** was identified as a leading compound with submicromolar activity, and structural optimization yielded **6y**, which exhibited the highest potency (MIC_50_: 0.36 µM for ATCC25923; 0.02 µM for USA300 JE2) and the greatest efficacy window (~885). In an H460 lung epithelial infection model mimicking MRSA pneumonia, **6y** markedly reduced intracellular bacterial loads with minimal host cell damage, demonstrating superior activity to vancomycin and efficacy comparable to linezolid.

Given their structural similarity to 3-acyl-2-phenylamino-1,4-dihydroquinolin-4-one (APDQ) derivatives, which have been shown to disrupt peptidoglycan biosynthesis (11), it is plausible that these compounds may also interfere with bacterial cell wall synthesis. However, the precise antibacterial mechanism of the 2-(amino)quinazolin-4(3*H*)-one derivatives, including **6l** and **6y,** remains under investigation, and ongoing mechanistic studies are expected to provide further insight.

Collectively, these findings establish **6l** as a key parental compound and **6y** as a lead candidate for further development toward novel therapeutics targeting MRSA infections.

## Figures and Tables

**Table 1:** Antibiotic effects of 2-(amino)quinazolin-4(3*H*)-one derivatives.

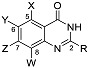										
No	Compound	X	Y	Z	W	R	ATCC25923 MIC_50_ (μM) ^a^	USA300 MIC_50_ (μM) ^a^	HepG2 IC_50_ (μM) ^b^	Efficacy Window (IC_50 HepG2_/MIC_50 USA300_)
1	**1**	Cl	H	H	Cl	3,5-Cl_2_-PhNH-	3.9	12.9	-	-
2	**6a**	H	H	Cl	Cl	3,5-Cl_2_-PhNH-	10.8	6.9	-	-
3	**6b**	H	H	Morpholine	H	3,5-Cl_2_-PhNH-	>100	>100	-	-
4	**6c**	H	OMe	OMe	H	3,5-Cl_2_-PhNH-	>100	>100	-	-
5	**6d**	H	H	OMe	H	3,5-Cl_2_-PhNH-	>100	>100	-	-
6	**6e**	H	H	NO_2_	H	3,5-Cl_2_-PhNH-	3.0	1.6	9.3	5.9
7	**6f**	H	Br	H	Br	3,5-Cl_2_-PhNH-	6.0	1.2	9.6	8.3
8	**6g**	H	F	F	H	3,5-Cl_2_-PhNH-	4.0	1.4	12.9	8.9
9	**6h**	H	Cl	Cl	H	3,5-Cl_2_-PhNH-	3.7	0.3	10.2	30.3
10	**6i**	H	Cl	H	Cl	3,5-Cl_2_-PhNH-	5.7	1.8	39.5	21.8
11	**6j**	H	H	H	H	3,5-Cl_2_-PhNH-	5.9	1.9	29.7	15.7
12	**6k**	H	H	CF_3_	H	3,5-Cl_2_-PhNH-	1.3	0.3	10.5	39.0
13	**6l**	H	H	Cl	H	3,5-Cl_2_-PhNH-	1.0	0.6	62.2	101.3
14	**6m**	H	H	Cl	H	PhNH-	>100	>100	-	-
15	**6n**	H	H	Cl	H	3-F-PhNH-	4.5	3.0	19.7	6.6
16	**6o**	H	H	Cl	H	2,4-F_2_-PhNH-	>100	>100	-	-
17	**6p**	H	H	Cl	H	3-MeO-PhNH-	35.1	24.9	-	-
18	**6q**	H	H	Cl	H	3,5-F_2_-PhNH-	2.1	0.9	35.5	39.2
19	**6r**	H	H	Cl	H	3-CF_3_-PhNH-	3.1	2.2	15.1	6.9
20	**6s**	H	H	Cl	H	CyclohexymethylNH-	35.5	18.1	-	-
21	**6t**	H	H	Cl	H	2-Cl-PhNH-	>100	>100	-	-
22	**6u**	H	H	Cl	H	3-Cl-PhNH-	3.5	1.1	4.3	4.0
23	**6v**	H	H	Cl	H	4-Cl-PhNH-	2.7	1.7	25.5	15.0
24	**6w**	H	H	Cl	H	3,5-F_2_-PhCH_2_NH-	>100	>100	-	-
25	**6x**	H	H	Cl	H	2,4-Cl_2_-PhCH_2_NH-	3.4	1.7	15.3	8.8
26	**6y**	H	H	Cl	H	3,4-F_2_-PhCH_2_NH-	0.36	0.02	20.2	885.2
Erythromycin							0.7	0.49	>100	-
Vancomycin							0.3	0.35	>100	-

^a^ MIC_50_ values for the synthesized compounds were derived from the results of at least two independent experiments against ATCC25923 or USA300 JE2 strains. ^b^ IC_50_ values for the synthesized compounds were derived from the results of at least two independent experiments using HepG2 cells.

**Table 2:** Antibiotic effects of 2-(amino)quinazolin-4(3*H*)-one derivatives in the cell infection model.

Compound	H460 Assay MIC_50_ (μM) ^a^	Efficacy Window (IC_50 HepG2_/MIC_50 USA300_)
**6e**	68.8	0.1
**6f**	8.7	1.1
**6g**	25.3	0.5
**6h**	4.1	2.5
**6i**	26.5	1.5
**6j**	35.4	0.8
**6k**	6.0	1.7
**6l**	12.1	5.2
**6n**	43.8	0.4
**6q**	8.7	4.1
**6r**	35.8	0.4
**6x**	17.8	0.9
**6u**	19.2	0.2
**6v**	23.9	1.1
**6y**	1.9	10.6
Vancomycin	7.7	
Erythromycin	0.1	

^a^ MIC_50_ values for the synthesized compounds were derived from the results of at least two independent experiments in the H460 infection model using the USA300 JE2 strain.

## Data Availability

The original contributions presented in this study are included in the article/Supplementary Materials. Further inquiries can be directed to the corresponding author(s).

## Supplementary Materials

The following supporting information can be downloaded at https://www.mdpi.com/article/10.3390/antibiotics14100967/s1: Figure S1: The structures of 2-(amino)quinazolin-4(3*H*)-one derivatives; Figure S2: ^1^H-NMR Spectrum (400 MHz, (CD_3_)_2_SO) and ^13^C-NMR Spectrum (100 MHz, (CD_3_)_2_SO) of compound **6f**; Figure S3: ^1^H-NMR Spectrum (400 MHz, (CD_3_)_2_SO) and ^13^C-NMR Spectrum (100 MHz, (CD_3_)_2_SO) of compound **6h**; Figure S4: ^1^H-NMR Spectrum (400 MHz, (CD_3_)_2_SO) and ^13^C-NMR Spectrum (100 MHz, (CD_3_)_2_SO) of compound **6j**.; Figure S5: ^1^H-NMR Spectrum (400 MHz, (CD_3_)_2_SO) and ^13^C-NMR Spectrum (100 MHz, (CD_3_)_2_SO) of compound **6k**; Figure S6: ^1^H-NMR Spectrum (400 MHz, (CD_3_)_2_SO) and ^13^C-NMR Spectrum (100 MHz, (CD_3_)_2_SO) of compound **6l**; Figure S7: ^1^H-NMR Spectrum (400 MHz, (CD_3_)_2_SO) and ^13^C-NMR Spectrum (100 MHz, (CD_3_)_2_SO) of compound **6p**; Figure S8: ^1^H-NMR Spectrum (400 MHz, (CD_3_)_2_SO) and ^13^C-NMR Spectrum (100 MHz, (CD_3_)_2_SO) of compound **6q**; Figure S9: ^1^H-NMR Spectrum (400 MHz, (CD_3_)_2_SO) and ^13^C-NMR Spectrum (100 MHz, (CD_3_)_2_SO) of compound **6t**; Figure S10: ^1^H-NMR Spectrum (400 MHz, (CD_3_)_2_SO) and ^13^C-NMR Spectrum (100 MHz, (CD_3_)_2_SO) of compound **6w**; Figure S11: ^1^H-NMR Spectrum (400 MHz, (CD_3_)_2_SO) and ^13^C-NMR Spectrum (100 MHz, (CD_3_)_2_SO) of compound **6y**; Figure S12. The UPLC-MS spectrum of compound **6f**; Figure S13. The UPLC-MS spectrum of compound **6h**; Table S1. Results of hERG binding assay and cytotoxicity of **6l** and **6y**; Table S2. Result of microsomal stability, plasma protein binding rate, and CYP inhibition of **6l** and **6y**; Table S3. Rat pharmacokinetic study of **6l** and **6y**.
