# Supplementary material for: Synthesis and Structure–Activity Relationship Study of 2-(amino)quinazolin-4(3H)-one Derivatives as Potential Inhibitors of Methicillin-Resistant Staphylococcus aureus (MRSA)
Source: Antibiotics (Basel). 2025 Sep 25;14(10):967. doi: 10.3390/antibiotics14100967 (PMC12561453; doi:10.3390/antibiotics14100967)
Supplement: Supplementary file 1 [file antibiotics-14-00967-s001.zip › antibiotics-3856400-supplementary.pdf]

Supplementary Materials

Synthesis and Structure–Activity Relationship Study of 2-(amino)quinazolin-4(3*H*)-one Derivatives as Potential Inhibitors of Methicillin-Resistant *Staphylococcus aureus* (MRSA)

Jun Young Lee [1,2,†], Hyunjung Lee [3,†], Sungmin Kim [1], Jihwan Gim [1], Yunmi Lee [3], Chae Jo Lim [4,5], Hyun-Seob Song [6,7], Hyeung-geun Park [2,*], Soojin Jang [3,*] and Chul Min Park [1,5,*]

1 Infectious Diseases Therapeutic Research Center, Korea Research Institute of Chemical Technology, 141 Gajeong-ro, Yuseong-gu, Daejeon 34114, Republic of Korea; ljy3695@krict.re.kr (J.Y.L.); ksm0191@krict.re.kr (S.K.); kimjh0063g@naver.com (J.G.)

2 Research Institute of Pharmaceutical Sciences, College of Pharmacy, Seoul National University, Seoul 08826, Republic of Korea

3 Antibacterial Resistance Laboratory, Institut Pasteur Korea, Seongam-si 13488, Republic of Korea; hyunjung.lee@ip-korea.org (H.L.); yunmi.lee@ip-korea.org (Y.L.)

4 Data Convergence Drug Research Center, Korea Research Institute of Chemical Technology, Daejeon 34114, Republic of Korea; chemlcj@krict.re.kr

5 Medicinal Chemistry and Pharmacology, Korea University of Science and Technology (UST), 141 Gajeong-ro, Yuseong-gu, Daejeon 34114, Republic of Korea

6 Department of Biological Systems Engineering, University of Nebraska-Lincoln, Lincoln, NE 68588, USA; hsong5@unl.edu

7 Department of Food Science and Technology, Nebraska Food for Health Center, University of Nebraska-Lincoln, Lincoln, NE 68588, USA

* Correspondence: hgpk@snu.ac.kr (H.-g.P.); soojin.jang@ip-korea.org (S.J.); parkcm@krict.re.kr (C.M.P.); Tel.: +82-2-880-7871 (H.-g.P.); +82-31-8081-8194 (S.J.); +82-42-860-7137 (C.M.P.)

† These authors contributed equally to this work.

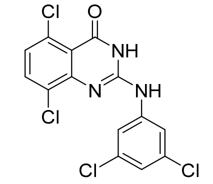

1

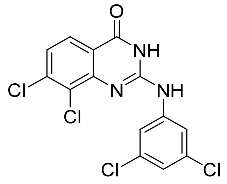

6a

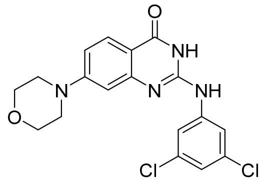

6b

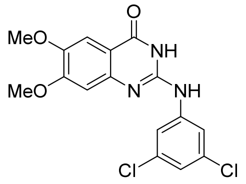

6c

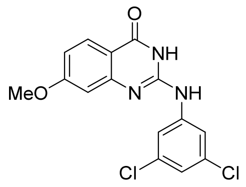

6d

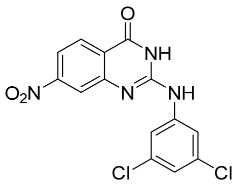

6e

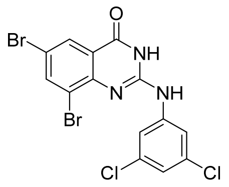

6f

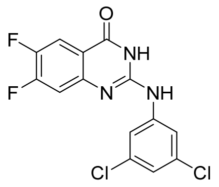

6g

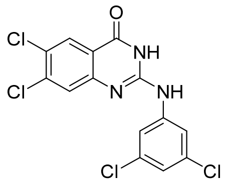

6h

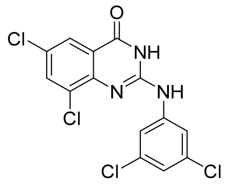

6i

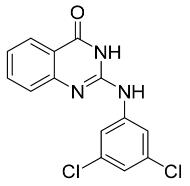

6j

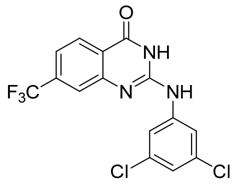

6k

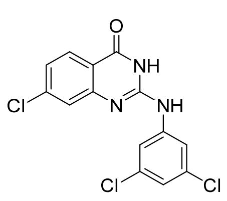

6l

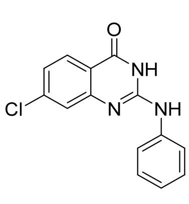

6m

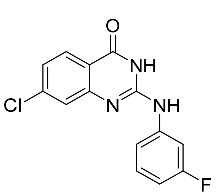

6n

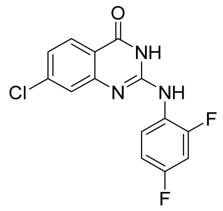

6o

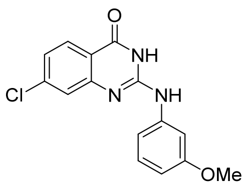

6p

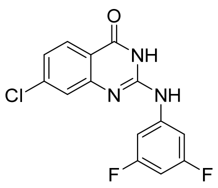

6q

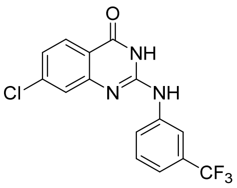

6r

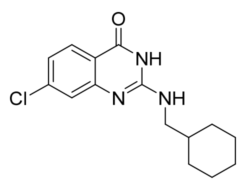

6s

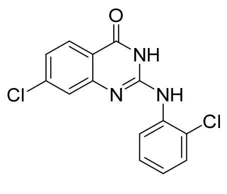

6t

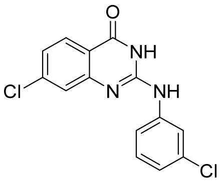

6u

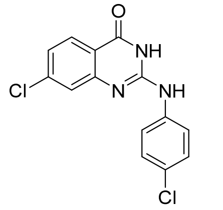

6v

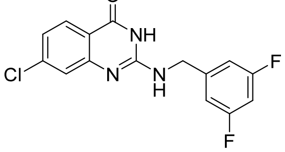

6w

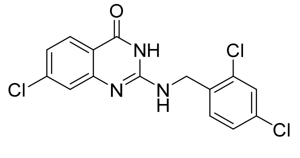

6x

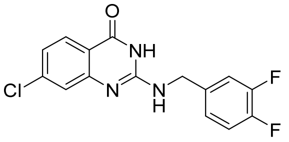

6y

Figure S1: The structures of 2-(amino)quinazolin-4(3*H*)-one derivatives.

^{1}H&^{13}C NMR Spectra of synthesized compounds (**Figure S2-Figure S11**)

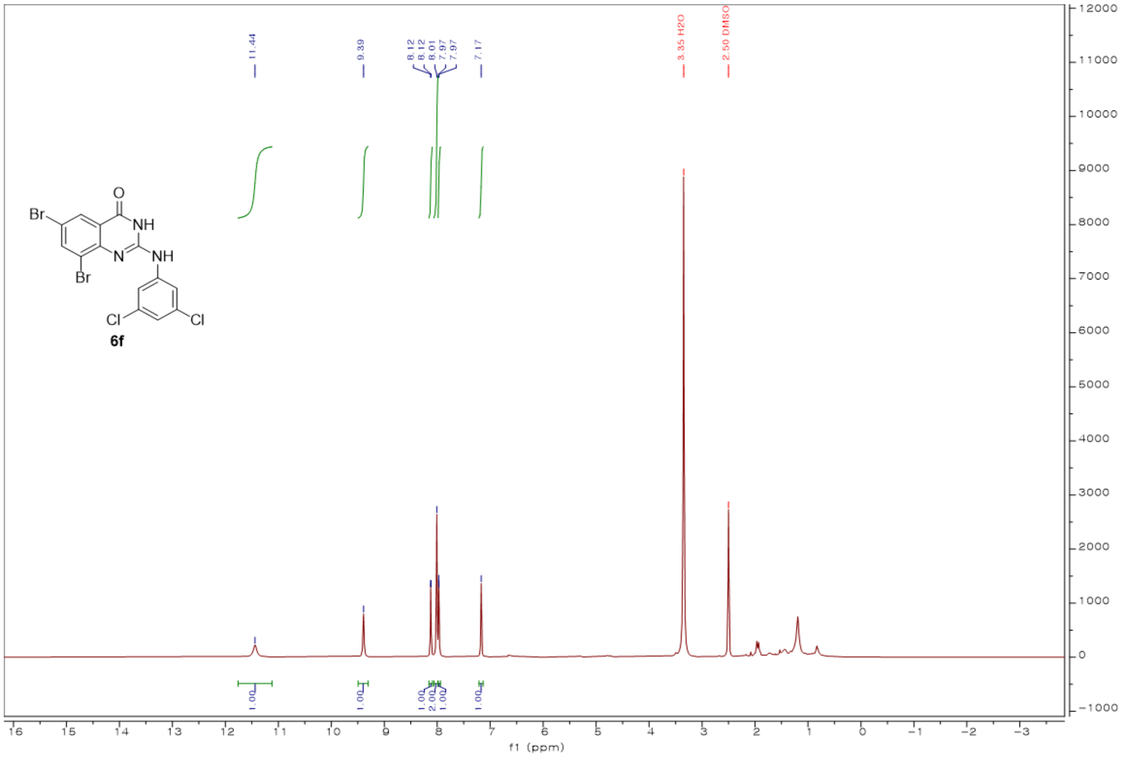

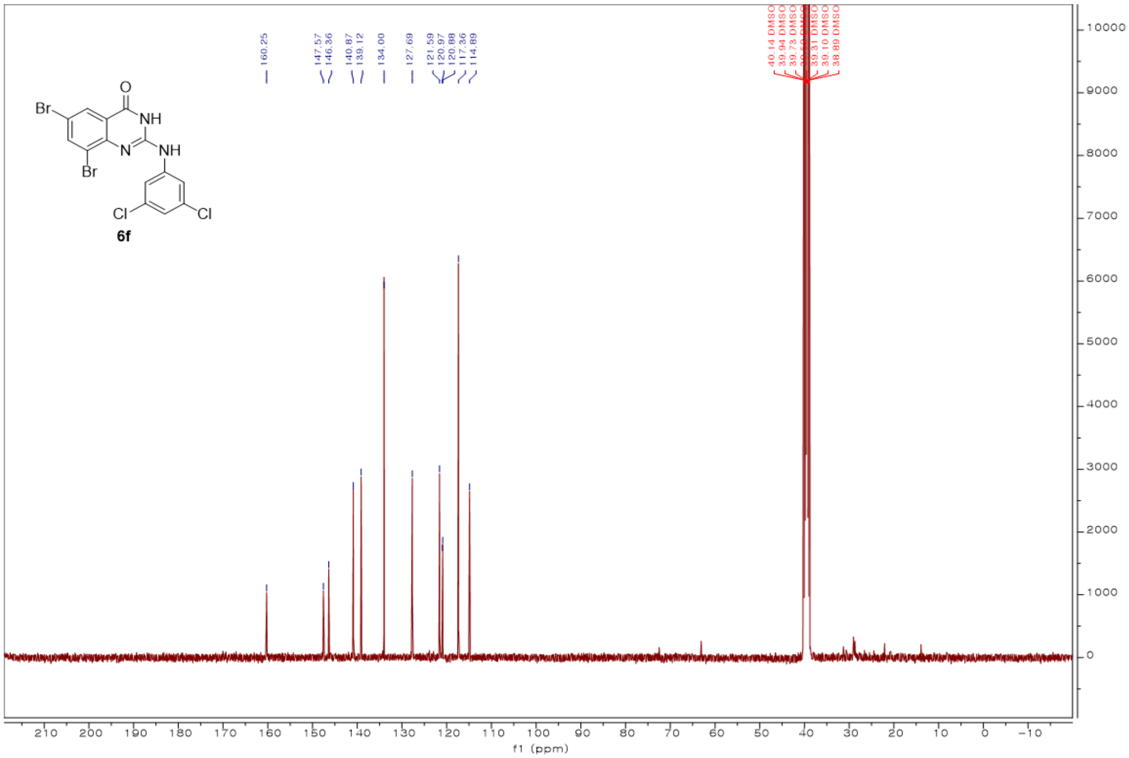

Figure S2: ^{1}H-NMR Spectrum (400 MHz, $(CD_3)_2SO$) and ^{13}C-NMR Spectrum (100 MHz, $(CD_3)_2SO$) of compound **6f**

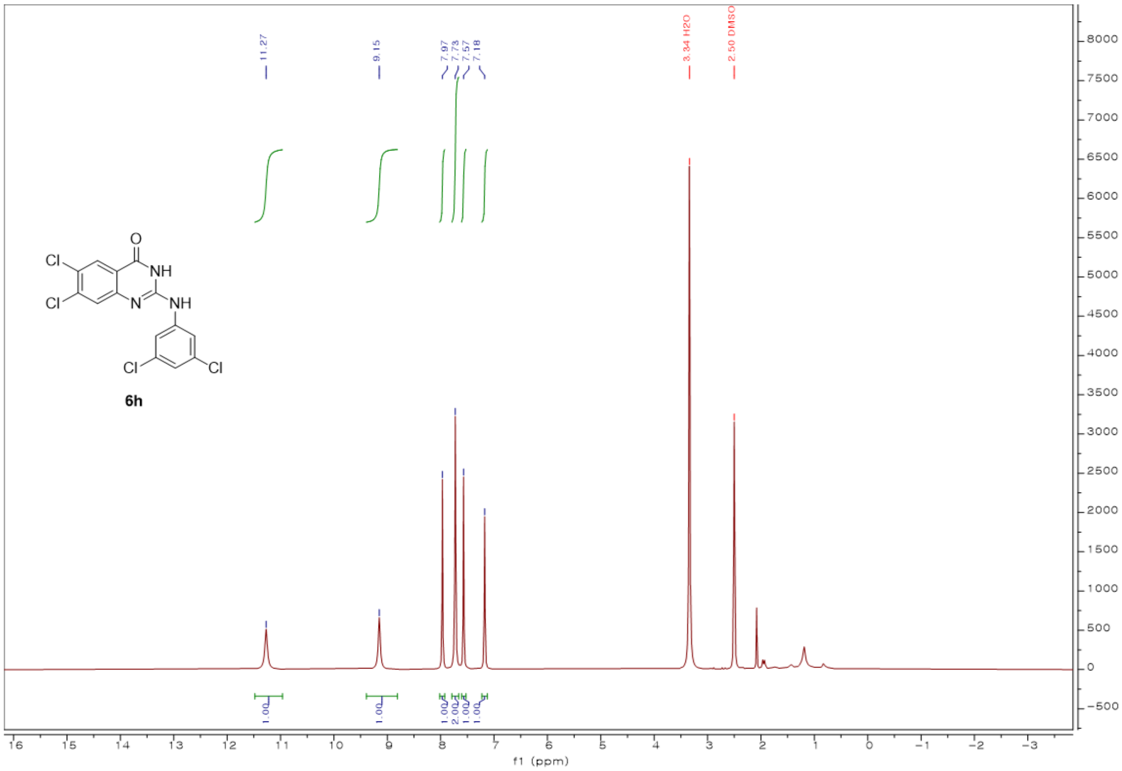

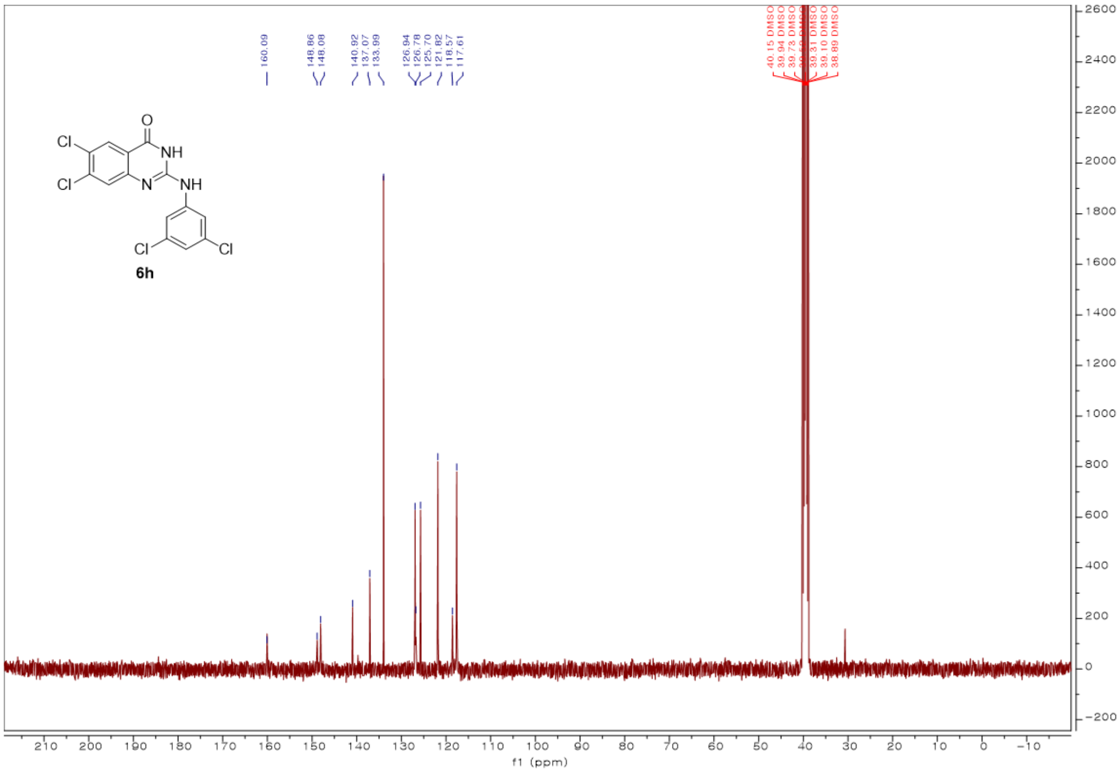

Figure S3: ^{1}H-NMR Spectrum (400 MHz, $(CD_3)_2SO$) and ^{13}C-NMR Spectrum (100 MHz, $(CD_3)_2SO$) of compound **6h**

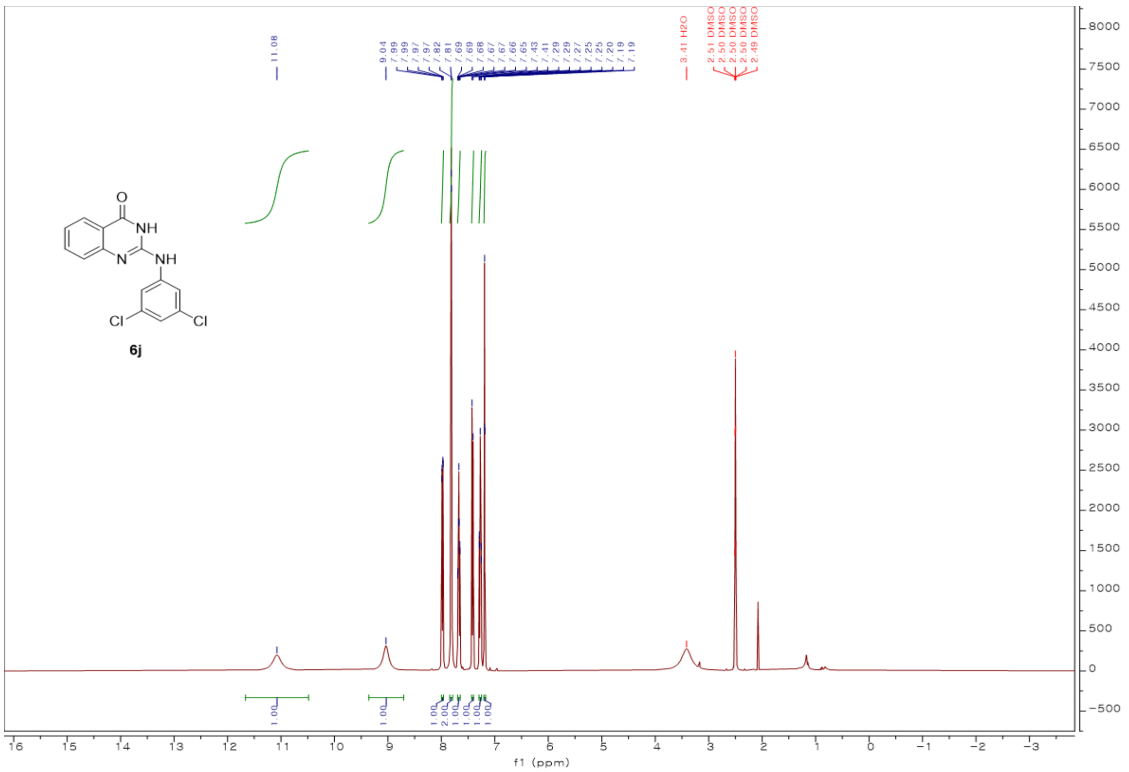

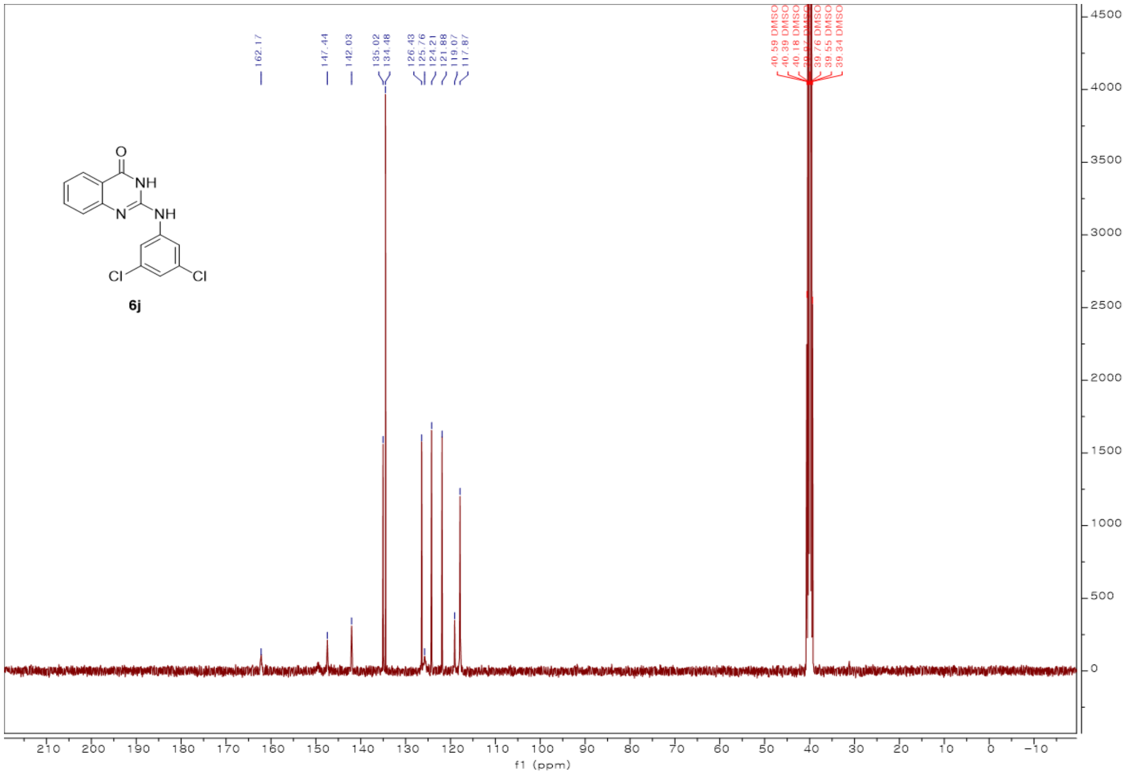

Figure S4: ^{1}H-NMR Spectrum (400 MHz, $(CD_3)_2SO$) and ^{13}C-NMR Spectrum (100 MHz, $(CD_3)_2SO$) of compound **6j**

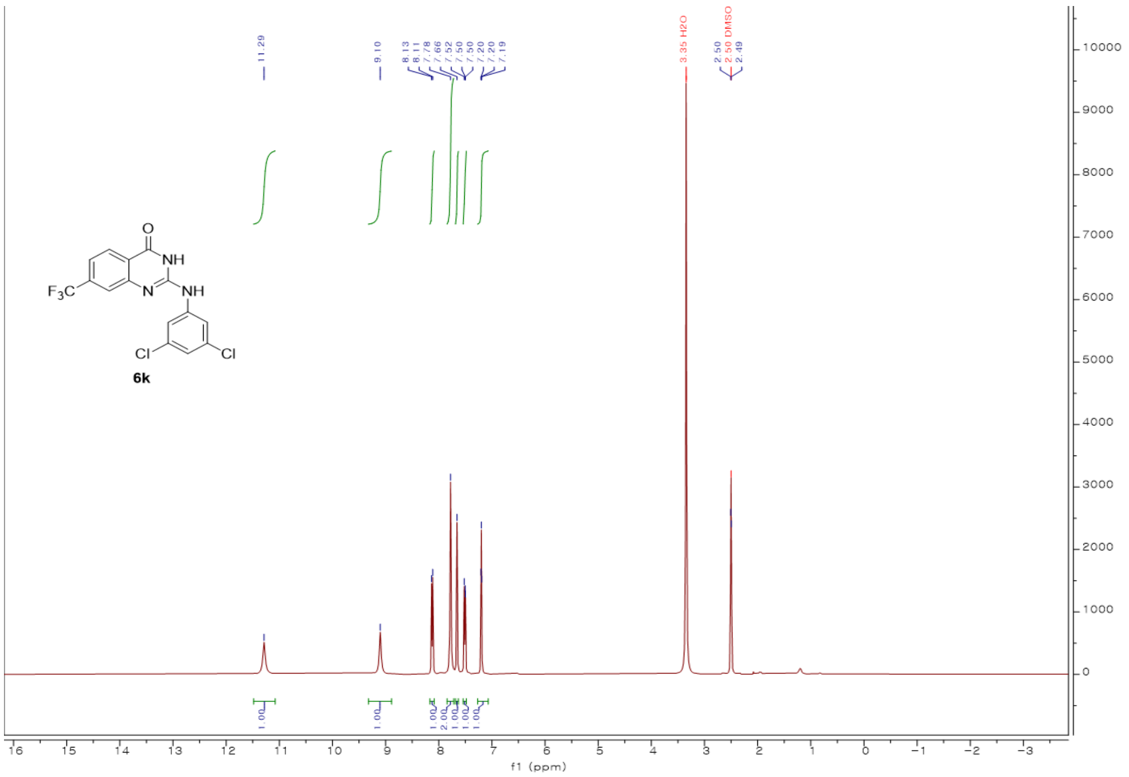

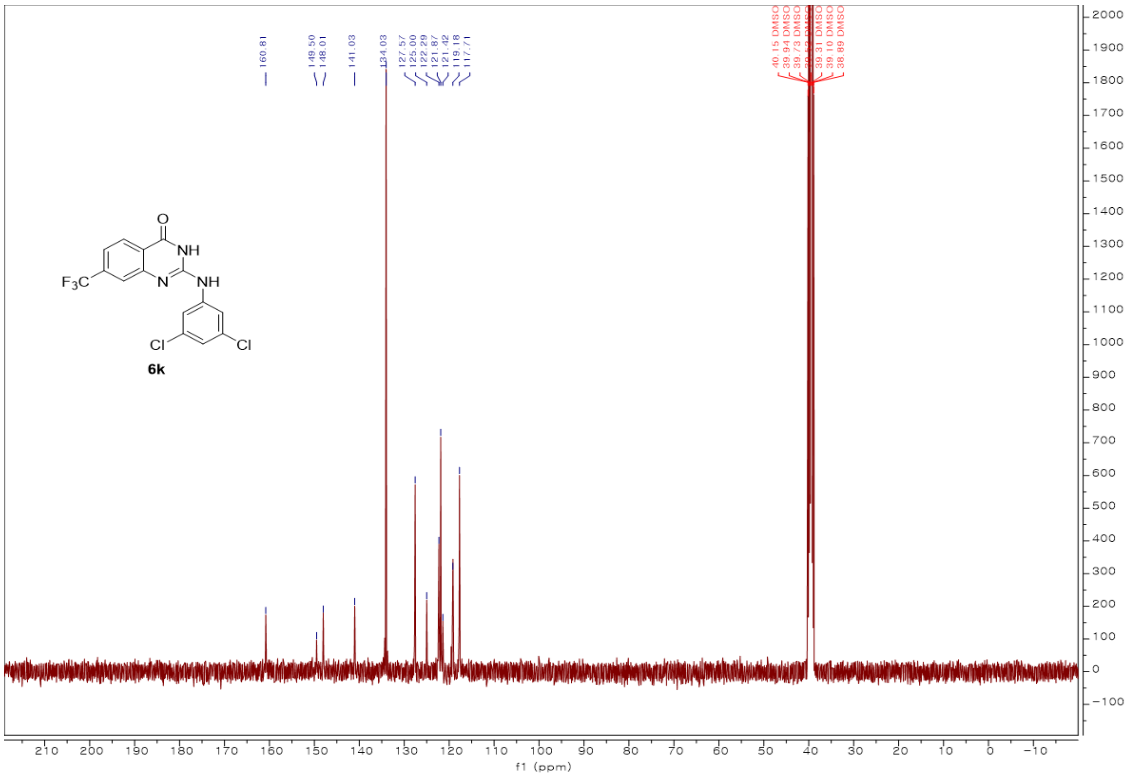

Figure S5: ^{1}H-NMR Spectrum (400 MHz, $(CD_3)_2SO$) and ^{13}C-NMR Spectrum (100 MHz, $(CD_3)_2SO$) of compound **6k**

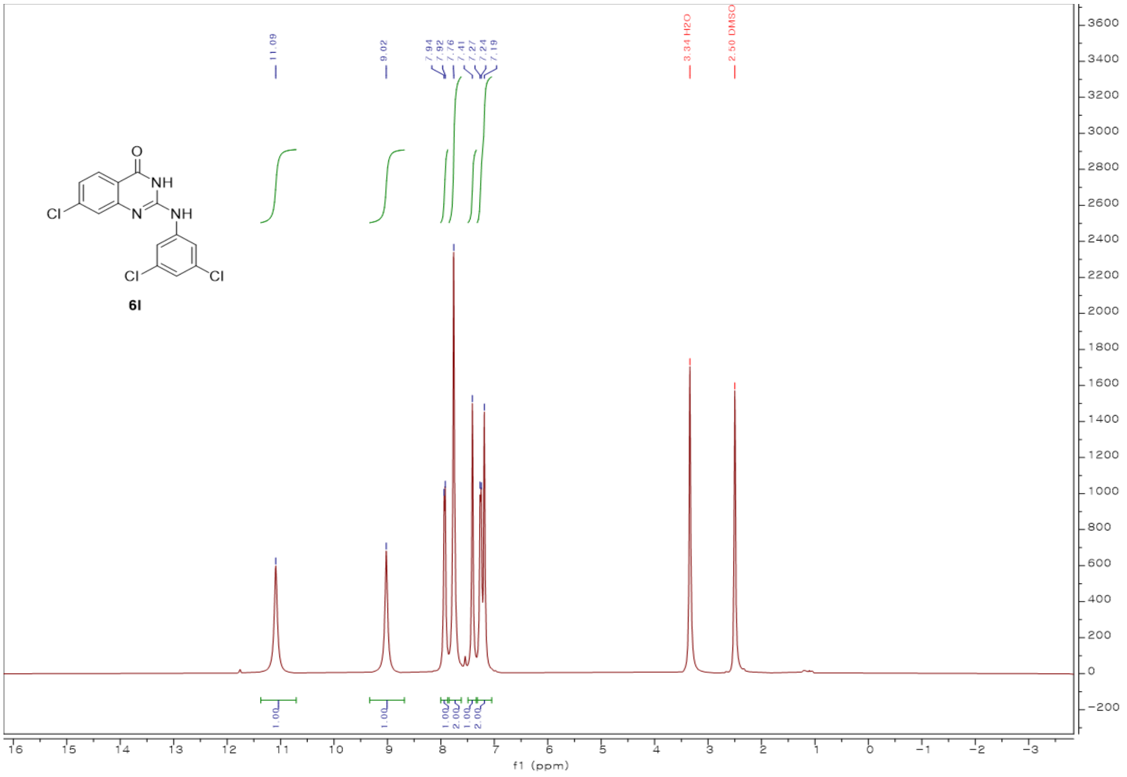

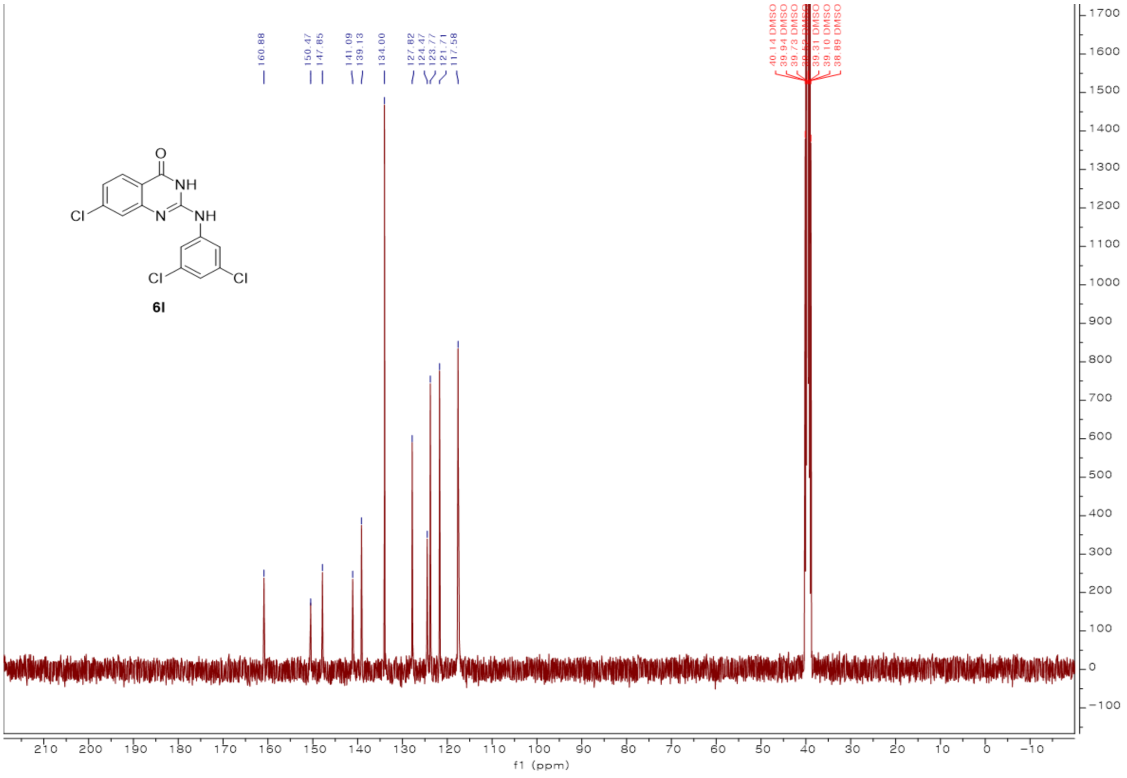

Figure S6: ^{1}H-NMR Spectrum (400 MHz, $(CD_3)_2SO$) and ^{13}C-NMR Spectrum (100 MHz, $(CD_3)_2SO$) of compound **6l**

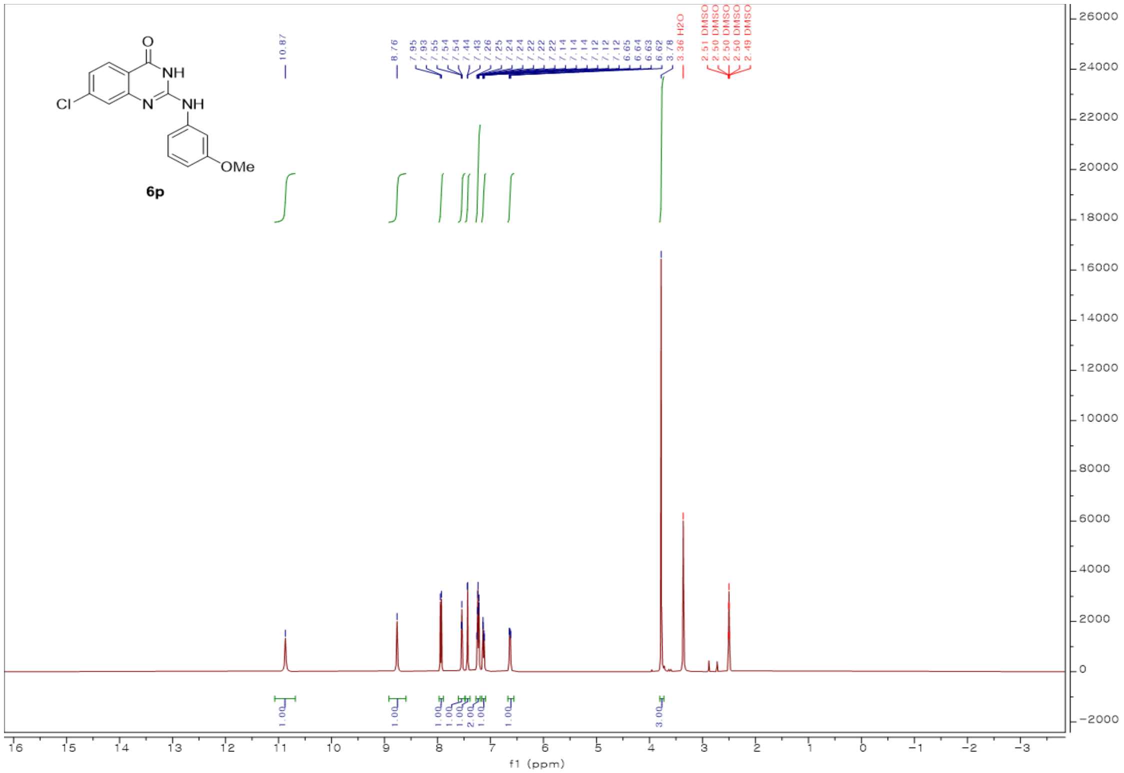

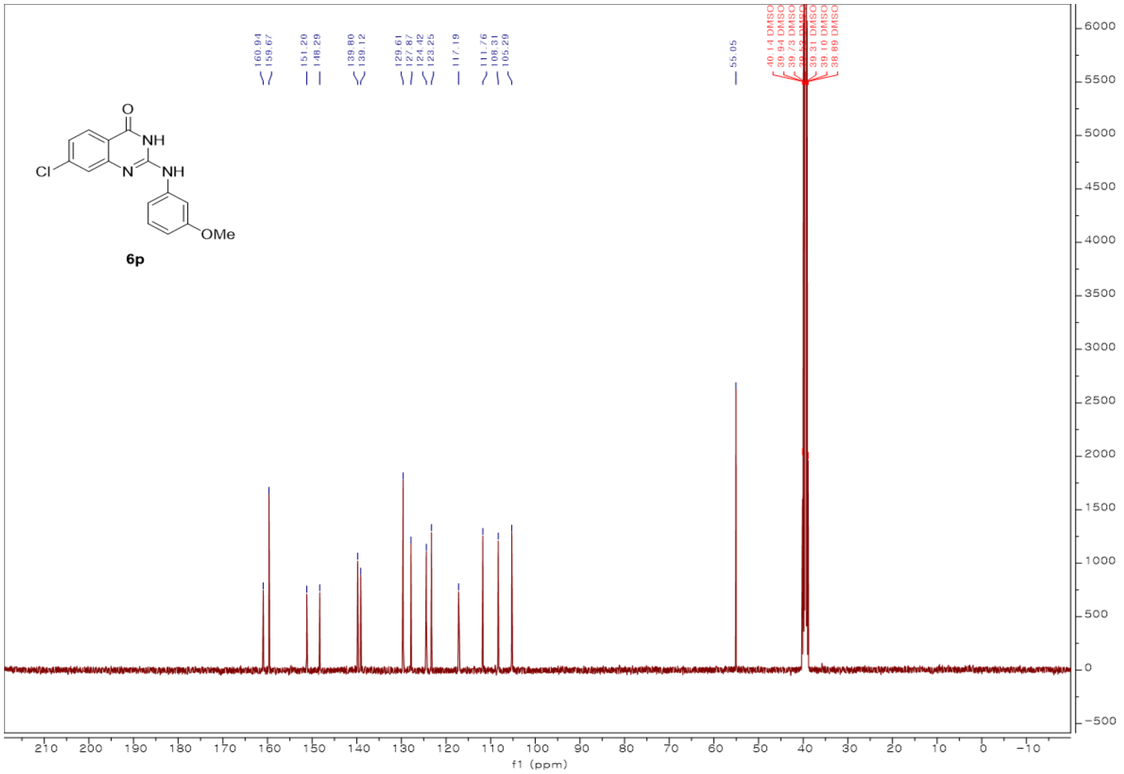

Figure S7: ^{1}H-NMR Spectrum (400 MHz, $(CD_3)_2SO$) and ^{13}C-NMR Spectrum (100 MHz, $(CD_3)_2SO$) of compound **6p**

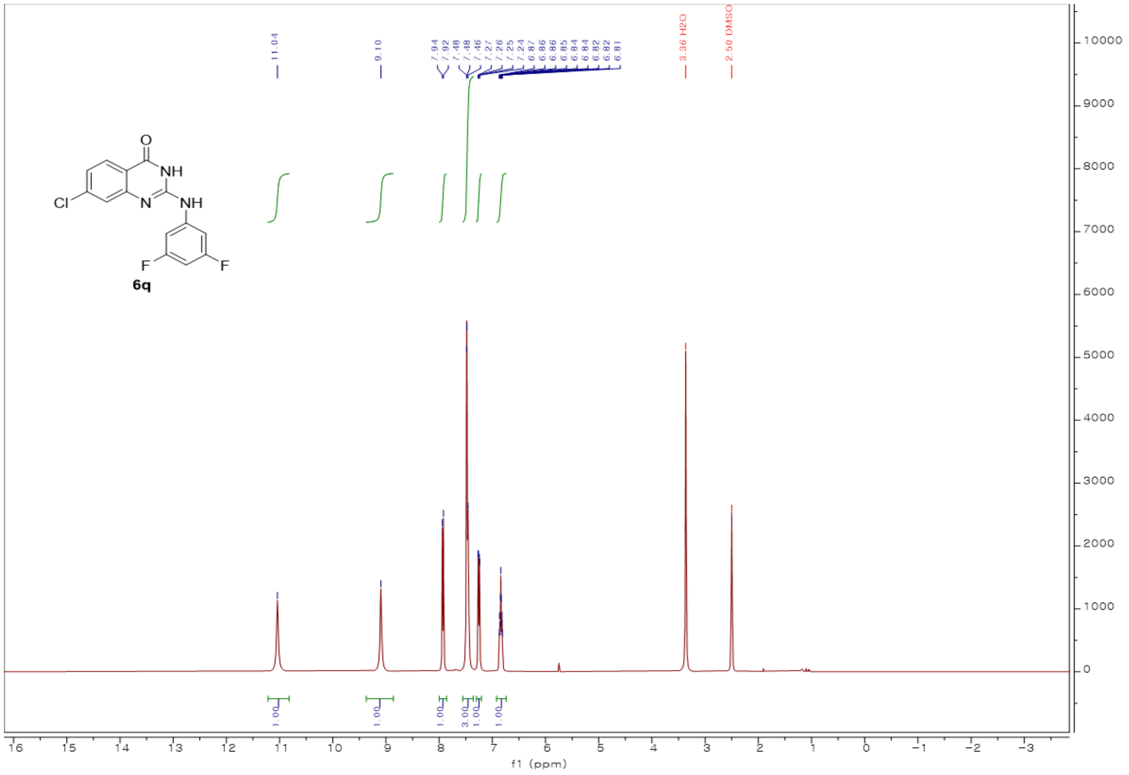

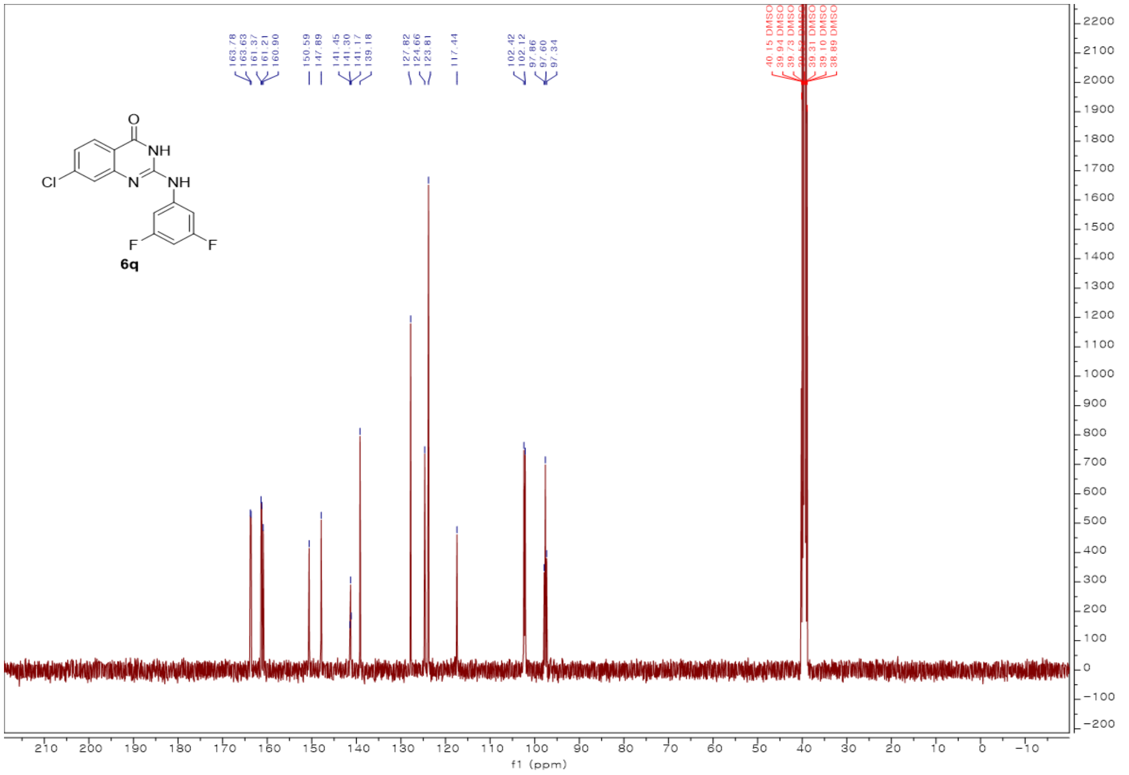

Figure S8: ^{1}H-NMR Spectrum (400 MHz, $(CD_3)_2SO$) and ^{13}C-NMR Spectrum (100 MHz, $(CD_3)_2SO$) of compound **6q**

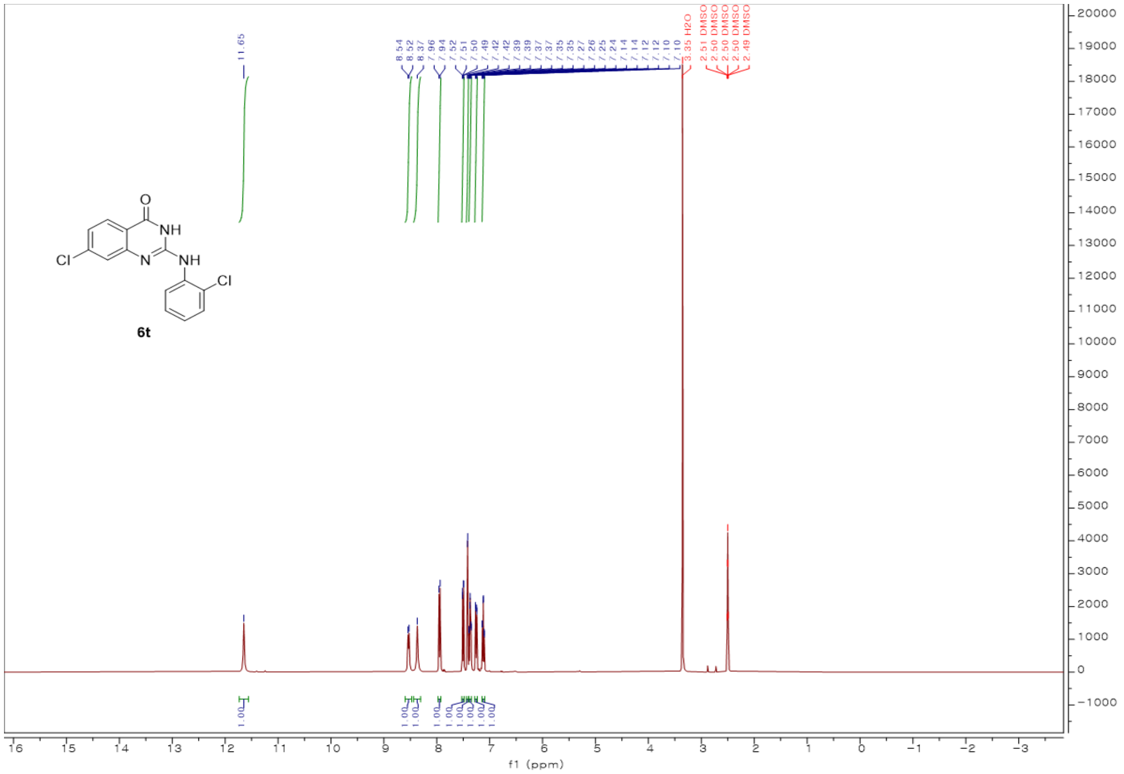

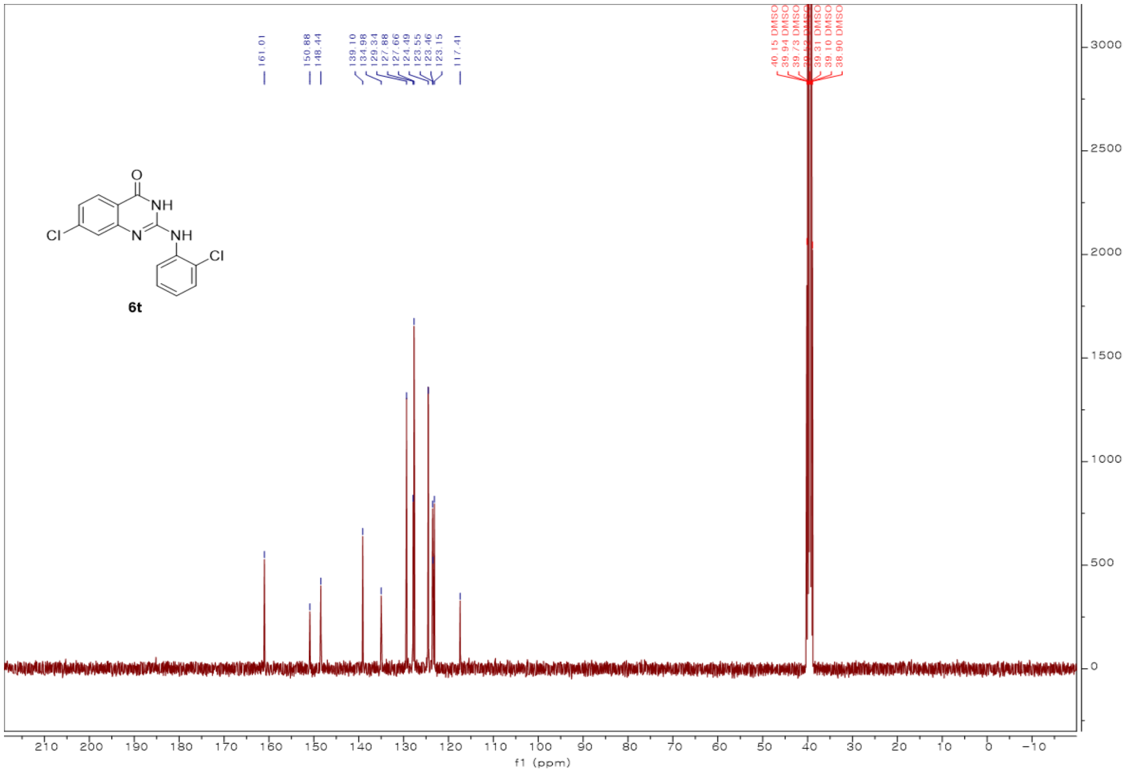

Figure S9: ^{1}H-NMR Spectrum (400 MHz, $(CD_3)_2SO$) and ^{13}C-NMR Spectrum (100 MHz, $(CD_3)_2SO$) of compound **6t**

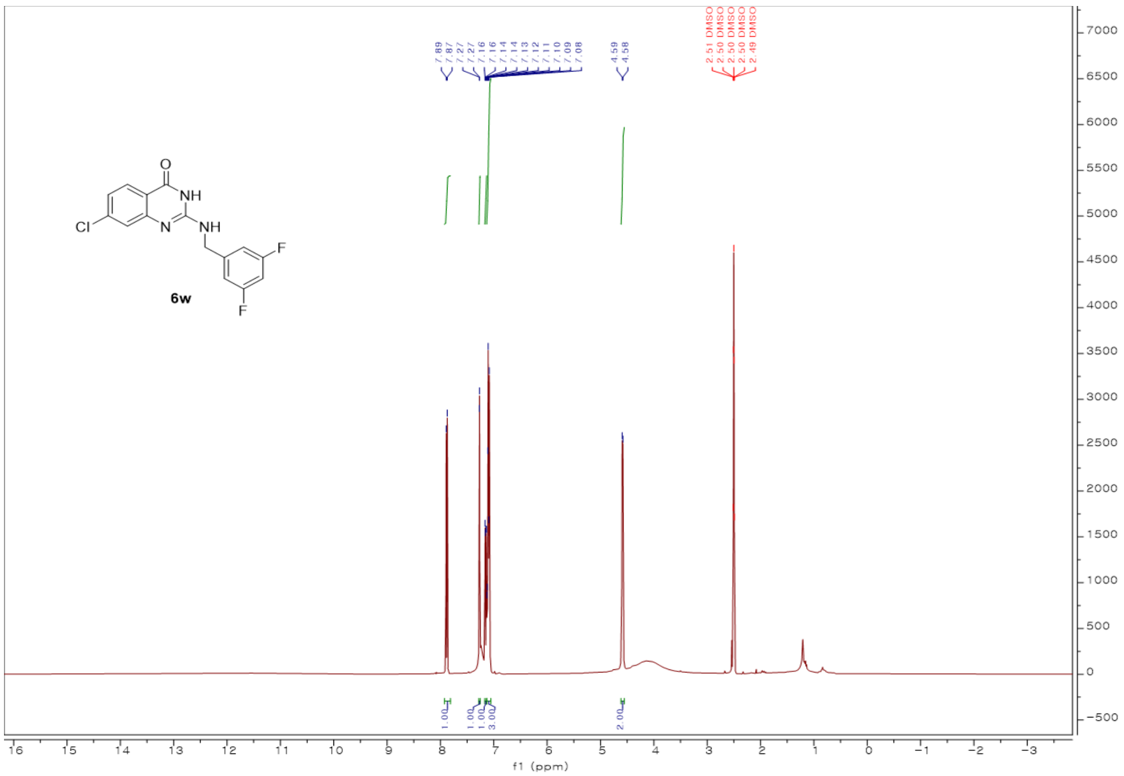

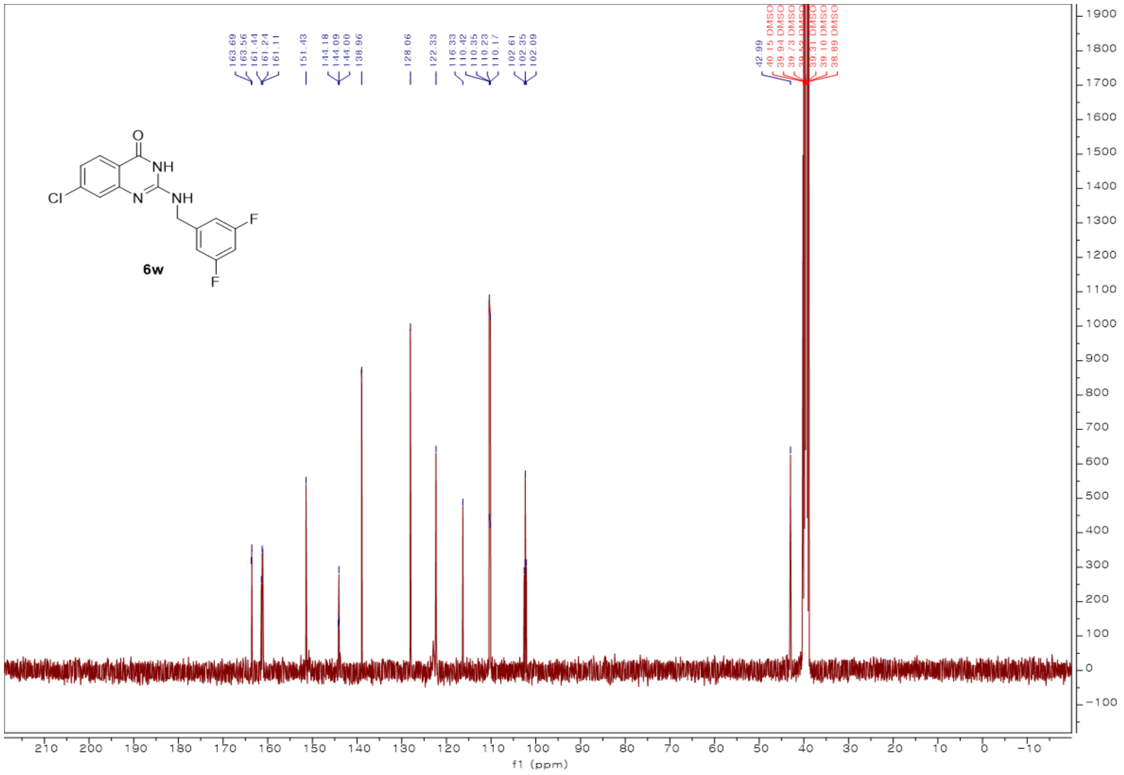

Figure S10: ^{1}H-NMR Spectrum (400 MHz, $(CD_3)_2SO$) and ^{13}C-NMR Spectrum (100 MHz, $(CD_3)_2SO$) of compound **6w**

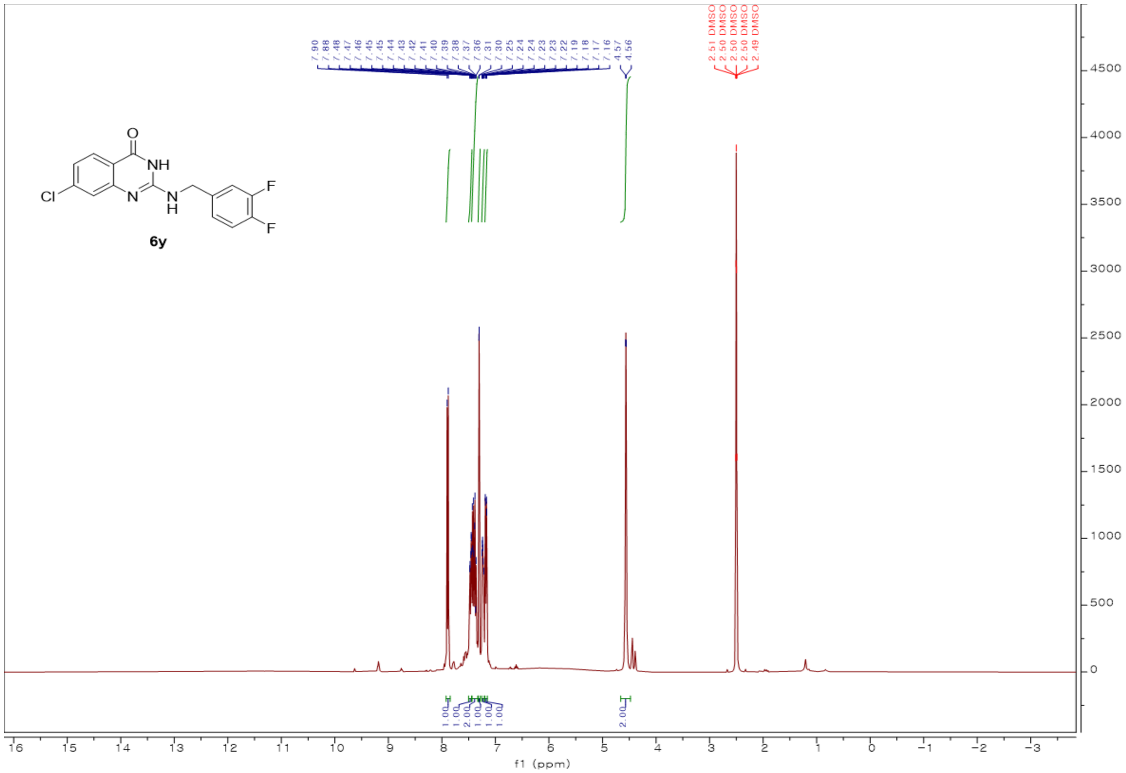

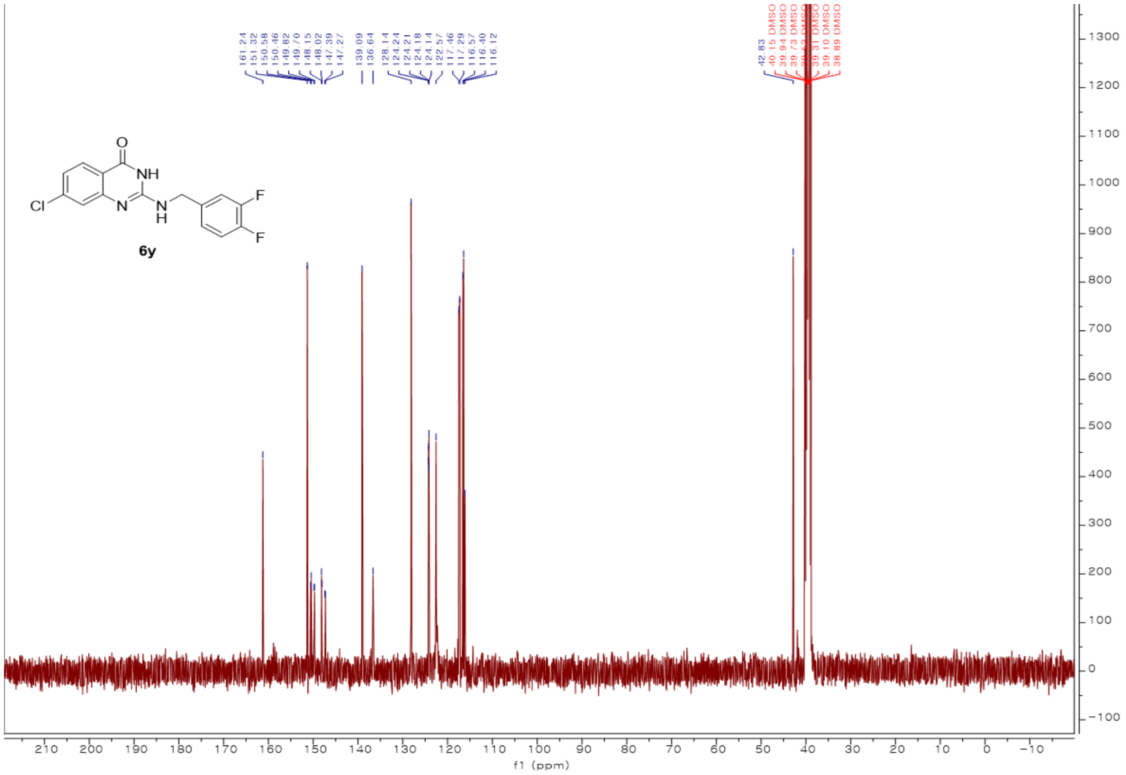

Figure S11: ^{1}H-NMR Spectrum (400 MHz, $(CD_3)_2SO$) and ^{13}C-NMR Spectrum (100 MHz, $(CD_3)_2SO$) of compound **6y**

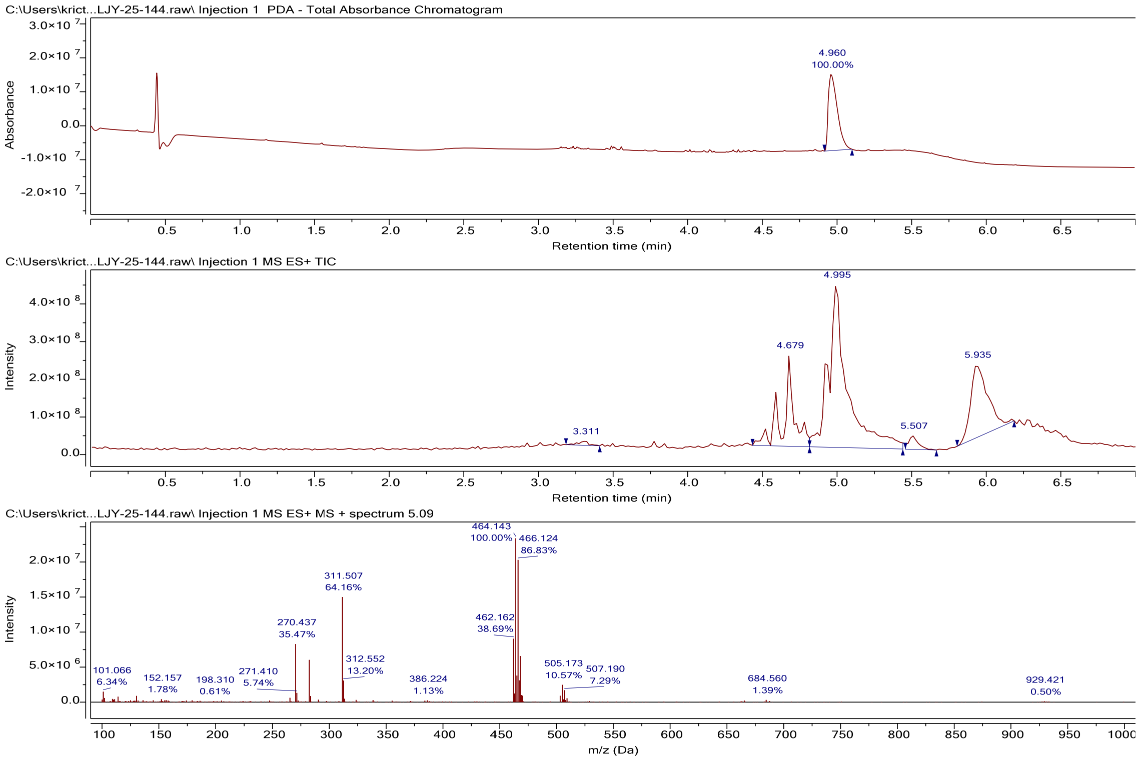

Figure S12. The UPLC-MS spectrum of compound **6f**

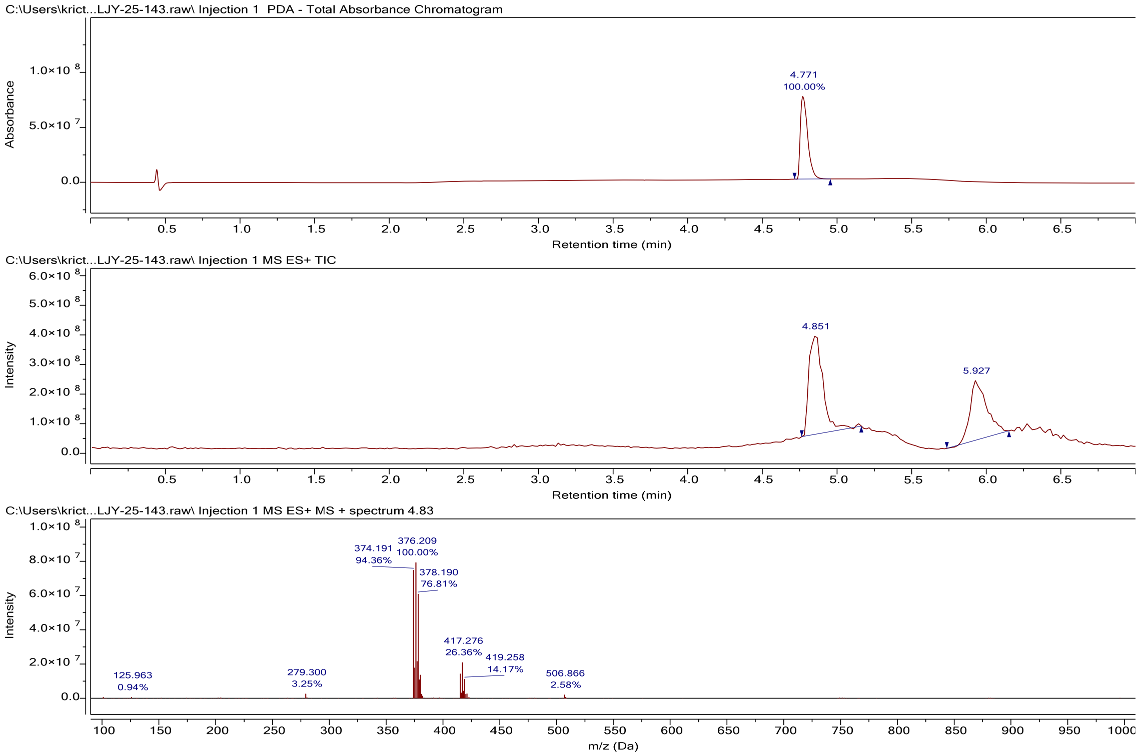

Figure S13. The UPLC-MS spectrum of compound **6h**

Table S1. Results of hERG binding assay and cytotoxicity of **6l** and **6y**

Compound	hERG[a]	Cytotoxicity[b]				
		VERO	HFL-1	L929	NIH 3T3	CHO-K1
**6l**	37.8	8.5	7.5	31.4	>100	11.0
**6y**	11.4	50.5	50.7	79.0	62.0	51.6

[a] % inhibition at 10 μM concentrations. [b] IC_{50} values in various mammalian cell lines. VERO: African green monkey kidney cell line, HFL-1: human embryonic lung cell line, L929: NCTC clone 929, mouse fibroblast cell line, NIH 3T3: mouse embryonic fibroblast cell line, CHO-K1: Chinese hamster ovary cell line.

Table S2. Result of microsomal stability, plasma protein binding rate, and CYP inhibition of **6l** and **6y**

Compound	MS[a]		PPB[b]		CYP inhibition[c]				
	rat	human	rat	human	1A2	2C9	2C19	2D6	3A4
**6l**	104.7	99.3	99.9	99.8	15.1	18.2	<1	20.1	32.2
**6y**	53.8	42.9	99.5	99.8	92.5	91.0	93.6	90.1	91.4

[a] % of remaining after 30 min. [b] plasma protein binding rate (%) at 5 μM concentrations. [c] % of CYP inhibition in human liver microsomes at 10 μM concentrations.

Table S3. Rat pharmacokinetic study of **6l** and **6y**

Compound	**6l**		**6y**	
Parameters[a]	I.V., 2 mg/kg	P.O., 10 mg/kg	I.V., 5 mg/kg	P.O., 10 mg/kg
T_{max} (h)	NA[b]	2.5	NA	5.3
C_{max} (μg/h)	NA	0.9	NA	0.2
$T_{1/2}$ (h)	2.2	5.7	8.5	3.6
AUC (μg·h/mL)	8.5	6.6	2.8	1.9
CL (L/h/kg)	0.25	NA	1.8	NA
V_{ss} (L/kg)	1.6	NA	5.2	NA
F_t (%)	NA	15.6	NA	33.4

[a] All results are mean of experiments using three male rats. [b] NA: not applicable
